# Portal hypertension contributes to ascites formation via the Piezo1−nuclear factor kappa-B−aquaporin1 pathway in liver cirrhosis

**DOI:** 10.1038/s12276-025-01554-6

**Published:** 2025-10-01

**Authors:** Ning Wei, Li Du, Zhuanglong Xiao, Lei Zhang, Yangyang Zhou, Haonan Gao, Minghui Liu, Chengbo Wang, Xiaohua Hou, Yan Li, Yuhu Song

**Affiliations:** 1 Department of Gastroenterology, Union Hospital, Tongji Medical College, Huazhong University of Science and Technology, Wuhan, China; 2 Department of Gastroenterology, The Central Hospital of Wuhan, Tongji Medical College, Huazhong University of Science and Technology, Wuhan, China; 3 Department of Gastroenterology, the First People’s Hospital of Yunnan Province, Affiliated Hospital of Kunming University of Science and Technology, Kunming, China

**Keywords:** Liver cirrhosis, Mechanisms of disease

## Abstract

Portal hypertension is critical to the development of ascites in liver cirrhosis. The aim of our study was to evaluate the contribution of portal hypertension to ascites formation and explore the underlying mechanism. Here, the role of portal hypertension in cirrhotic ascites was determined through a meta-analysis of portal pressure in cirrhotic patients and animal models. The mechanism underlying the involvement of portal hypertension in ascites formation was explored. The meta-analysis showed hepatic venous pressure gradient in patients with cirrhosis with ascites was significantly higher than that in patients with cirrhosis without ascites. In carbon tetrachloride (CCl_4_)-treated rats, portal pressure of cirrhotic rats with ascites (19.83 cmH_2_O) was significantly higher than that in cirrhotic rats without ascites (14.90 cmH_2_O). In a novel murine model created by thioacetamide (TAA)/CCl_4_ administration plus partial portal vein ligation (PPVL), a significant increase in portal pressure and ascites amount was observed in the TAA/CCl_4_ + PPVL group compared with the TAA/CCl_4_ group. In the mice treated with TAA/CCl_4_ plus PPVL, the amount of ascites decreased significantly in mice with endothelial deletion of Piezo1 (Piezo1^△EC^) compared with Piezo1^flox/flox^ mice. Finally, Piezo1 in liver sinusoidal endothelial cells and peritoneal endothelial cells promoted the development of portal hypertensive ascites via the nuclear factor kappa-B (NF-ĸB)−aquaporin1 (AQP1) pathway. In conclusion, Piezo1 in endothelium contributes to the formation of portal hypertensive ascites via the NF-ĸB−AQP1 pathway in liver cirrhosis, which indicates that blockage of the Piezo1−NFĸB−AQP1 pathway may be a useful strategy for the management of ascites in liver cirrhosis.

## Portal hypertension drives ascites formation in liver cirrhosis

Ascites, a fluid buildup in the abdomen, is a common sign of worsening liver cirrhosis and linked to lower survival rates. This study addresses the lack of a reliable animal model to explore how portal hypertension contributes to ascites. The researchers developed a new mouse model to study this. They used chemicals and partial portal vein ligation to mimic liver cirrhosis and ascites in mice. This model showed that increased portal pressure is crucial for ascites development. The study also explored how Piezo1, a protein that senses mechanical changes, might be involved. In liver cirrhosis, Piezo1 activation in endothelial cells may lead to ascites by increasing AQP1 expression, a protein that helps water move across cell membranes. The findings suggest that targeting the Piezo1−NF-κB−AQP1 pathway could be a new way to treat ascites in liver cirrhosis.

This summary was initially drafted using artificial intelligence, then revised and fact-checked by the author.

## Introduction

Ascites is commonly the first decompensation-defining event of liver cirrhosis and is associated with reduced survival in patients with liver cirrhosis^1–4^. The factors involved in ascites formation contain portal hypertension, sodium and water retention, hypoproteinemia, systemic inflammation and so on. In liver cirrhosis, portal hypertension is a critical event in the development of ascites^3,4^. To obtain more convincing evidence, a systemic review and meta-analysis in cirrhotic patients with or without ascites is required. Most importantly, the mechanism underlying the involvement of portal hypertension in ascites formation should be explored in a murine model, with a reproducible and reliable murine model with liver cirrhosis and ascites established. However, a suitable model has not been available until now, which confines research on the mechanism underlying the contribution of portal hypertension to ascites formation. Thus, a novel murine model with liver cirrhosis and ascites should be developed for exploring the pathogenesis.

Mechanotransduction, the conversion of a mechanical stimulus into a biological response, constitutes various physiological processes, such as touch perception, pain, proprioception, vascular development and blood pressure regulation. In liver cirrhosis, hepatic microcirculatory dysfunction and increased intrahepatic vascular resistance lead to elevated portal pressure, resulting in complications such as ascites. Accumulating evidence suggests that pathogenic mechanotransduction is involved in the pathogenesis of portal hypertension complications, and the identification of molecular targets involved in pathogenic mechanotransduction represent a novel therapeutic strategy^5–7^.

The Piezo family, a class of mechanosensitive cation channel proteins, plays a key role in various mechanotransduction processes^8^. Piezo1 occurs in endothelial cells and vascular smooth muscle cells^9^. Piezo1 in endothelial cells contributes to blood pressure regulation and vascular permeability^10^. Additionally, a recent study found that elevated pressure in lung microvessels induced Piezo1 activation, which resulted in leaky lung microvessels and edema formation^11^. In liver cirrhosis, peritoneal microvascular endothelial cells/liver sinusoidal endothelial cells (LSECs) are constantly under hydrostatic pressure due to portal hypertension^12^, which probably activates Piezo channels in endothelial cells. Subsequently, Piezo activation in endothelial cells/LSEC may lead to the development of ascites.

Here, we assessed the contribution of portal hypertension to ascites formation and explored the underlying mechanism in liver cirrhosis. First, a systemic review and meta-analysis was performed to evaluate the difference in hepatic venous pressure gradient (HVPG) between patients with cirrhosis with ascites and patients with cirrhosis without ascites. Then, animal models with liver cirrhosis and ascites demonstrated the essential contribution of portal hypertension to ascites formation in liver cirrhosis. Finally, the mechanism underlying the contribution of portal hypertension to ascites formation was explored through murine models and cell experiments.

## Materials and methods

### Meta-analysis

Detailed materials and methods are provided in the Supplementary Information. A meta-analysis was performed to determine the relation between portal pressure and ascites formation in patients with cirrhosis. The published studies on HVPG in patients with cirrhosis with or without ascites were retrieved through PubMed, EMBASE, the Web of Science and Cochrane Library databases.

### Animal model of liver cirrhosis with ascites

A rat model of liver cirrhosis with ascites was established with subcutaneous injection of carbon tetrachloride (CCl_4_) for 17 weeks after receiving phenobarbital (0.3 g/l in drinking water) for 1 week. A novel murine model of liver cirrhosis with ascites was established through the administration of chemicals (thioacetamide (TAA) or CCl_4_) followed by partial portal vein ligation (PPVL). Then, animal models of liver cirrhosis were evaluated by gross pathology, hematoxylin and eosin (HE) staining, Sirius red staining, liver functional tests, ascites amount, portal pressure and immunostaining.

### Underlying mechanism of portal hypertension involved in the formation of ascites

The mice with endothelial cell-specific deletion of Piezo1 (Piezo1^△EC^) were generated through breeding Cdh5-CreERT2 mice^13^ with Piezo1-floxed mice (Piezo1^fl/fl^). Then, Piezo1^△EC^ mice were treated with TAA/CCl_4_ administration plus PPVL, which generated cirrhotic mice with ascites. The role of Piezo1 in portal hypertensive ascites was evaluated by ascites amount, portal pressure and histological examination. To investigate the mechanism of Piezo1 involved in portal hypertensive ascites, we analyzed data of human umbilical vein endothelial cells (HUVECs) exposed to laminar flow shear stress from comprehensive gene expression databases. In addition, the mechanism underlying the contribution of Piezo1 to portal hypertensive ascites was explored through quantitative PCR with reverse transcription (RT−qPCR), chromatin immunoprecipitation (ChIP)−qPCR, immunostaining, calcium imaging and the blockage of gene expression.

## Results

### Portal pressure increases in patients with cirrhosis with ascites

**Fig. 1:**
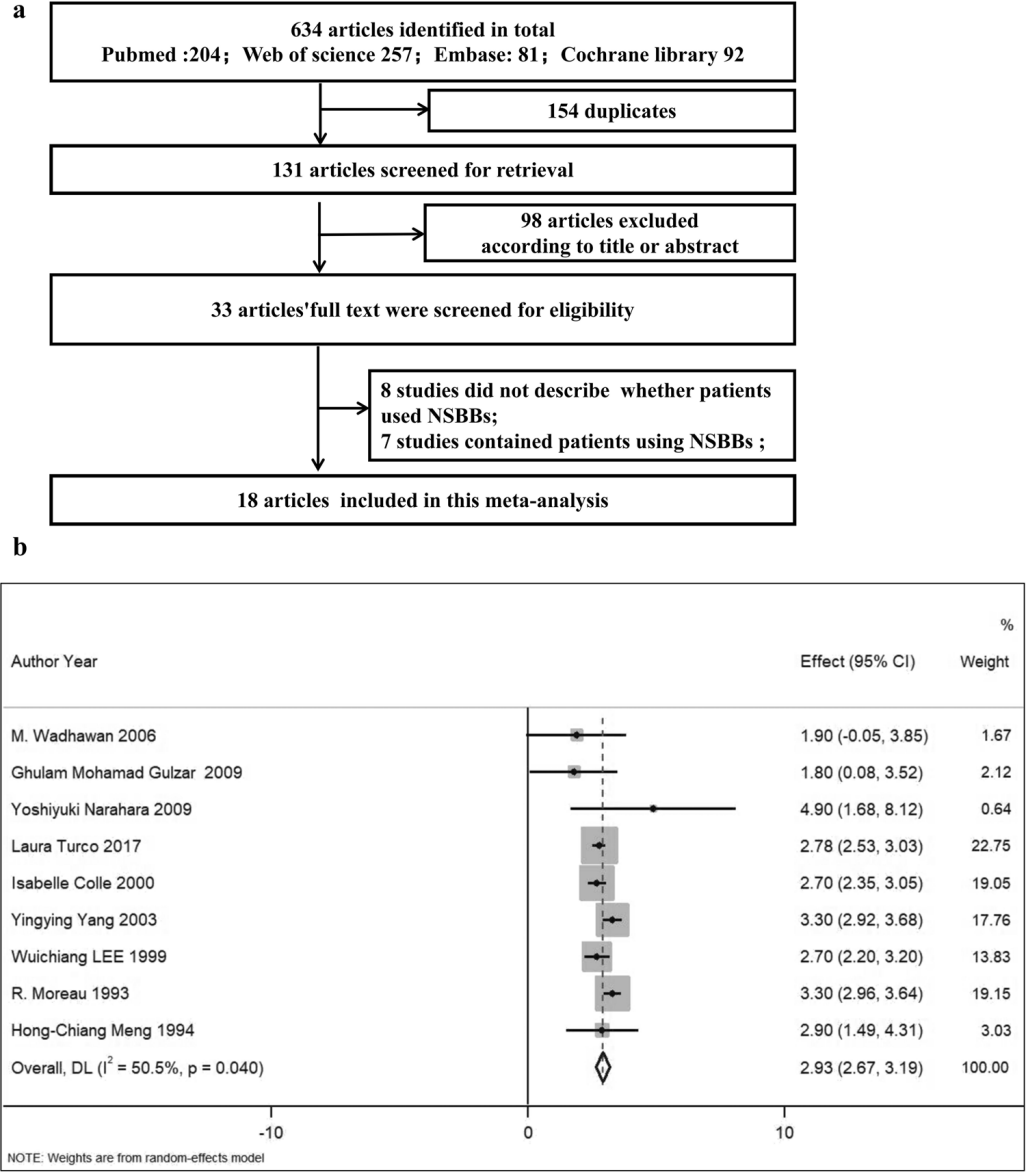
Flow chart of study inclusion in the meta-analysis and the result of the meta-analysis. **a** A flow chart of the study inclusion for the meta-analysis of HVPG in patients with cirrhosis with or without ascites. **b** HVPG measurements in patients with cirrhosis with or without ascites from the meta-analysis. NSBBs, nonselective or nonspecific beta blockers.

Portal hypertension is critical to the formation of cirrhotic ascites. To confirm the key role of portal hypertension, a systematic review and meta-analysis was conducted to evaluate the correlation between portal hypertension and ascites formation in liver cirrhosis. Eighteen eligible studies involving patients with cirrhosis with HVPG measurements were included (Fig. 1a and Supplementary Table 1)^14–31^. As expected, the meta-analysis demonstrated that portal pressure in patients with cirrhosis with ascites was significantly higher than that in patients with cirrhosis without ascites (Fig. 1b). The meta-analysis also showed that HVPG measurements were 14.95 mmHg, 18.66 mmHg and 20.81 mmHg in patients with cirrhosis without ascites, with ascites and with refractory ascites, respectively^32^, which indicates that portal pressure increased gradually with the development of ascites. These date reveal increased portal pressure in patients with cirrhosis with ascites.

### Increased portal pressure was observed in cirrhotic rats with ascites

To confirm the crucial role of portal hypertension, a rat model with liver cirrhosis and ascites was established through the administration of CCl_4_ (Supplementary Fig. 1a). After 18 weeks administration of CCl_4_, all survival rats had liver cirrhosis and 37.50% (6/16) of survival rats developed ascites (Supplementary Fig. 1b). Significant differences in fibrotic area, portal pressure and serum albumin (ALB) concentration were observed between cirrhotic rats with and without ascites (Supplementary Fig. 1c–e), while no significant differences were observed for serum alanine aminotransferase (ALT) or aspartate aminotransferase (AST) between the two groups (Supplementary Fig. 1f). Statistical analysis showed that portal pressure had a smaller *P* value than serum ALB concentration (Supplementary Fig. 1d, e). Collagen deposition is associated with liver stiffness, angiogenesis and contraction, which is closely related to portal hypertension^33^. These results reveal the importance of portal hypertension in the formation in cirrhotic ascites.

### A novel murine model with liver cirrhosis and ascites demonstrates the essential role of portal hypertension in ascites formation

**Fig. 2:**
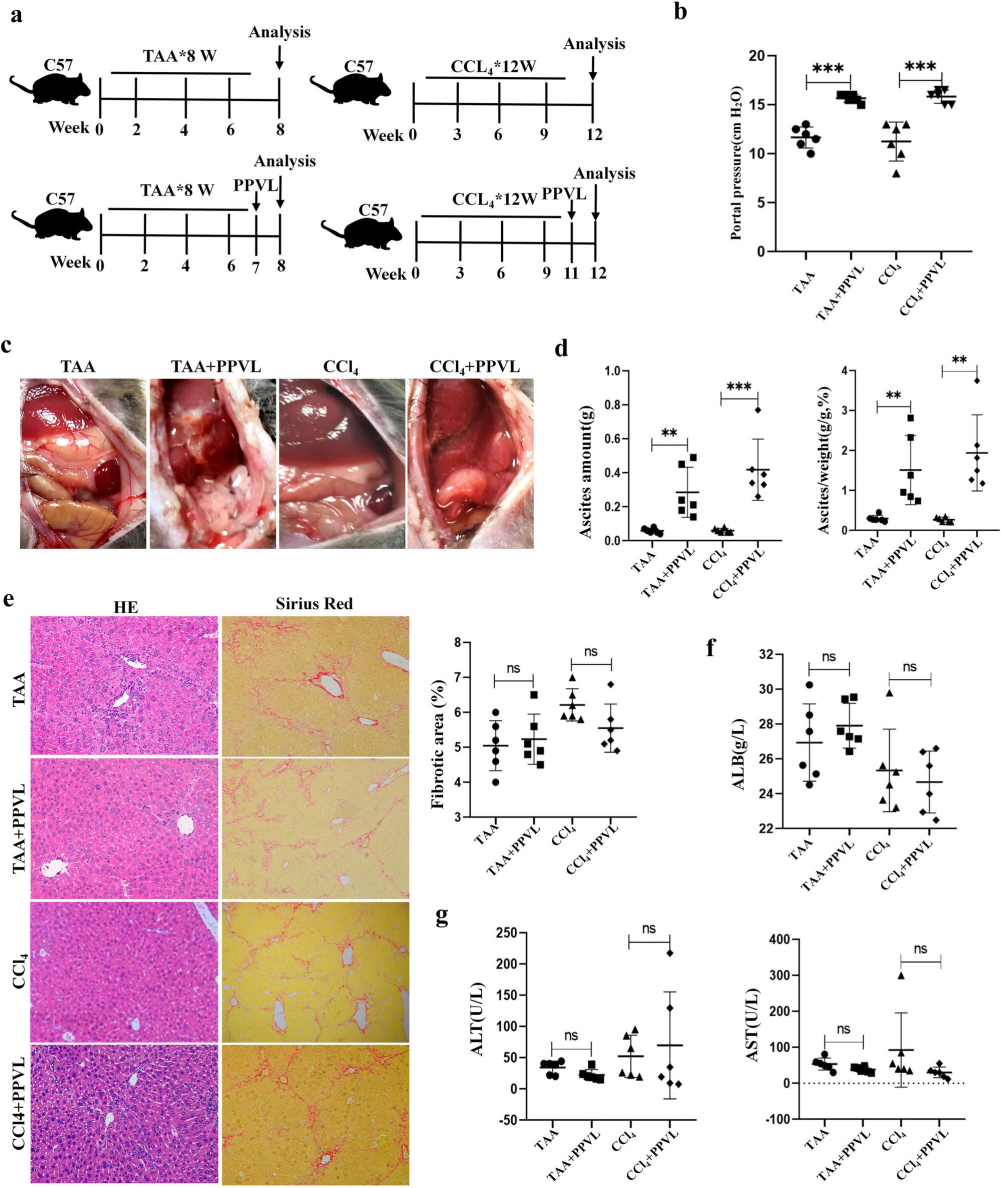
A novel murine model with liver cirrhosis and ascites revealing the crucial role of portal hypertension ascites formation. **a** The experimental protocol for murine model with liver cirrhosis and ascites, murine model with liver cirrhosis and ascites was established through the administration of TAA/CCl_4_ followed by PPVL. **b** Portal pressure in the mice treated with TAA/CCl_4_ or TTA/CCl_4_ + PPVL. **c** Representative macroscopic views of abdominal cavity showing the accumulation of peritoneal fluid in TTA/CCl_4_ + PPVL-treated mice. **d** The assessment of ascites amount in cirrhotic mice. Left: the amount of ascites. Right: the amount of ascites in body weight. **e** Representative images of hepatic HE staining and Sirius red staining from cirrhotic liver. Right: the percentage of fibrotic area was quantified by Sirius red staining, **f** Serum ALB concentration in cirrhotic mice. **g** Serum ALT and AST levels in cirrhotic mice. *P < 0.05, **P < 0.01 and ***P < 0.001. ns, not significant.

Rat models with liver cirrhosis and ascites are well established through CCl_4_ administration and bile-duct ligation. However, a reliable murine model with liver cirrhosis and ascites has not been established successfully until now. As described above, portal hypertension is essential to ascites formation. Our study showed that portal pressures in cirrhotic mice induced by TTA and CCl_4_ were 11.67 cmH_2_O and 11.25 cmH_2_O, respectively, which is lower than that in cirrhotic rats with ascites (19.83 cmH_2_O). To increase portal pressure, a novel murine model was created through TAA administration followed by PPVL (Fig. 2a). Around 90% of cirrhotic mice that received PPVL survived within a week after PPVL (Supplementary Table 5). The mice receiving TAA administration plus PPVL developed higher portal pressure compared with TAA-treated mice (15.67 cmH_2_O versus 11.67 cmH_2_O, *P* < 0.001) (Fig. 2b). As expected, the amount of ascites increased significantly in TAA plus PPVL-treated mice compared with TAA-treated mice (0.29 g versus 0.06 g, *P* < 0.01) (Fig. 2c, d). Additionally, 30% of the mice developed visible ascites (Fig. 2c, d and Supplementary Fig. 3). No significant differences in collagen deposition (Sirius red staining) and liver functional tests (serum ALB, ALT and AST) were observed between TAA-treated mice and TAA plus PPVL-treated mice (Fig. 2e−g). To confirm these results, a murine model with liver cirrhosis and ascites was also successfully created through CCl_4_ administration followed by PPVL. The mice receiving CCl_4_ administration plus PPVL developed higher portal pressure compared with CCl_4_-treated mice (15.67 cmH_2_O versus 11.25 cmH_2_O, *P* < 0.001) (Fig. 2b). The amount of ascites increased significantly in the CCl_4_ plus PPVL-treated mice compared with CCl_4_-treated mice (0.42 g versus 0.06 g, *P* < 0.001) (Fig. 2c, d), with 50% of mice developing visible ascites (Fig. 2c, d). No significant differences in collagen deposition and liver functional tests were observed between CCl_4_-treated mice and CCl_4_ plus PPVL-treated mice (Fig. 2e−g). These results indicate that increased portal pressure promotes ascites formation in cirrhotic mice though PPVL, demonstrating the essential role of portal hypertension in the development of ascites caused by liver cirrhosis.

### Piezo1 in endothelial cells promotes development of portal hypertensive ascites in liver cirrhosis

**Fig. 3:**
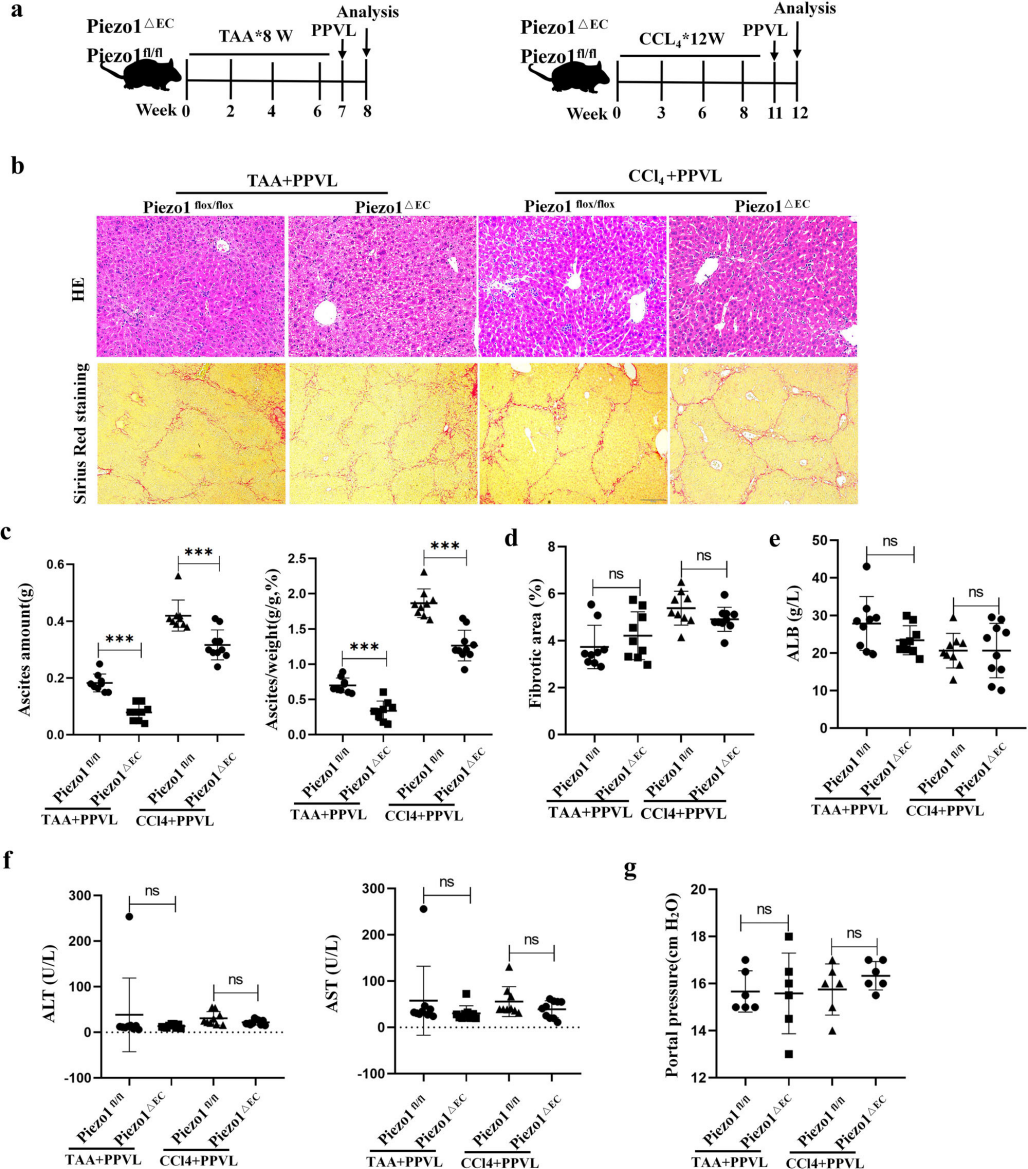
The critical role of endothelial Piezo1 in the development of portal hypertensive ascites using Piezo1^△EC^ mice and Piezo1^fl/fl^ mice. **a** The experimental protocol for cirrhotic mice with ascites using Piezo1^△EC^ mice and Piezo1^fl/fl^ mice. **b** Representative images of hepatic HE staining and Sirius red staining. **c** Evaluation of ascites in mice. Left: the amount of ascites. Right: the amount of ascites in body weight. **d** The percentage of fibrotic area was quantified by Sirius red staining. **e** Serum ALB concentration in mice. **f** Serum ALT and AST levels in mice. **g** Portal pressure in cirrhotic mice. *P < 0.05, **P < 0.01 and ***P < 0.001. ns, not significant.

Mechanosensitive ion channels are specialized transducers that convert mechanical force into electrochemical signals. Mechanosensitive ion channels are regulated by mechanical forces such as hydrostatic pressure, membrane stretch and shear stress. Thus, we hypothesized that portal hypertension induces the activation of mechanosensitive ion channels in endothelial cells. To simulate the pathological dysfunction of endothelial cells in portal hypertension, HUVECs were exposure to high hydrostatic pressure (Supplementary Fig. 4a) and the expression of mechanosensitive ion channels was determined. The results showed that Piezo1 expression was highest in HUVECs (Supplementary Fig. 4b). In addition, Fluo-3AM imaging demonstrated that high hydrostatic pressure induced calcium influx in HUVECs (Supplementary Fig. 4c), which indicates that high pressure probably induced Piezo1 activation and high expression in the endothelial cells of the portal venous system (Supplementary Fig. 5). Ascites are mainly produced by the liver capsule and peritoneum in cirrhotic rats^34^. In liver cirrhosis, both LSECs and peritoneal endothelial cells are subjected to mechanical pressure caused by portal hypertension^12^. Thus, we hypothesized that Piezo1 in the endothelial cells of peritoneum and LSECs was involved in the formation of portal hypertensive ascites. To test the hypothesis, Piezo1^△EC^ mice were generated (Supplementary Fig. 6a), with the successful establishment of Piezo1^△EC^ mice demonstrated through RT−qPCR (Supplementary Fig. 6b, c). Then, murine models with liver cirrhosis and ascites were constructed using Piezo1^△EC^ mice and Piezo1^flox/flox^ mice (Fig. 3a, b). In TAA plus PPVL-treated mice, the amount of ascites in Piezo1^△EC^ mice was lower than that in Piezo1^flox/flox^ mice (0.08 g versus 0.18 g, *P* < 0.001) (Fig. 3c). There were no significant differences in liver functional tests (serum ALT, AST and ALB) and collagen deposition (Sirius red staining) between Piezo1^△EC^ mice and Piezo1^flox/flox^ mice (Fig. 3d−f). Interestingly, no significant difference in portal pressure was observed between Piezo1^△EC^ mice and Piezo1^flox/flox^ mice (Piezo1^△EC^ versus Piezo1^flox/flox^: 15.58 cmH_2_O versus 15.67 cmH_2_O, *P* > 0.05) (Fig. 3g). Thus, the critical contribution of Piezo1 to the formation of ascites in liver cirrhosis is revealed. Then, a murine model with liver cirrhosis and ascites was also established through CCl_4_ administration and PPVL using Piezo1^△EC^ mice and Piezo1^flox/flox^ mice. Similar results were observed in Piezo1^△EC^ mice and Piezo1^flox/flox^ mice after CCl_4_ plus PPVL treatment (ascites amount: 0.32 g versus 0.42 g, *P* < 0.001; portal pressure: 16.33 cmH_2_O versus 15.75 cmH_2_O, *P* > 0.05) (Fig. 3a–g). These results demonstrate the critical role of Piezo1 in the development of portal hypertensive ascites.

### Piezo1 promotes development of cirrhotic ascites via the NF-ĸB−AQP1 pathway

**Fig. 4:**
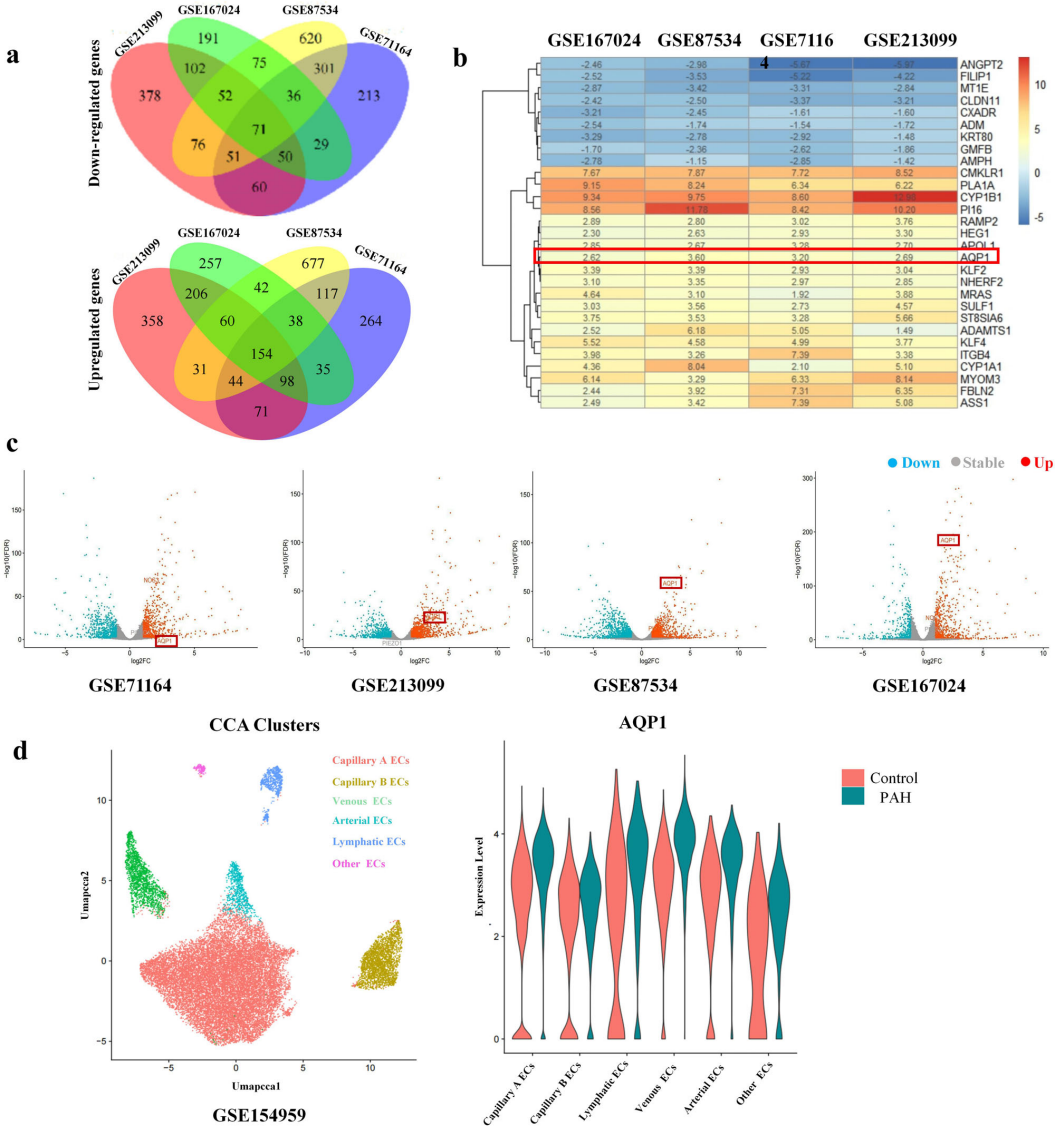
Portal hypertension induced AQP1 expression in peritoneal endothelium through Piezo1. **a** A Venn diagram of differentially expressed mRNA in HUVEC exposed to laminar shear flow. The expression profile database of HUVECs exposed to laminar shear stress was extracted from four GEO datasets. Top: the Venn diagram for downregulated genes. Bottom: the Venn diagram for upregulated genes. **b** A heat map generated by robust rank aggregation method representing the expression of significantly changed genes from four databases. The results show 9 downregulated genes and 20 upregulated genes, with upregulation of AQP1 in HUVECs exposed to laminar shear stress. **c** A volcano plot illustrating gene distributions based on expression levels, the results show that AQP1 expression increased in HUVECs exposed to laminar shear stress. **d** Single-cell sequencing showing high hydrostatic pressure induced AQP1 expression in the vascular endothelium of the lungs. Left: a cluster analysis divided endothelial cells into six subgroups. Right: increased AQP1 expression in endothelial cells subsets in a murine model of pulmonary hypertension. PAH Pulmonary Hypertension, Umapccal Uniform Manifold Approximation and Projection Clustering Calibration, EC Endothelial Cell.

**Fig. 5:**
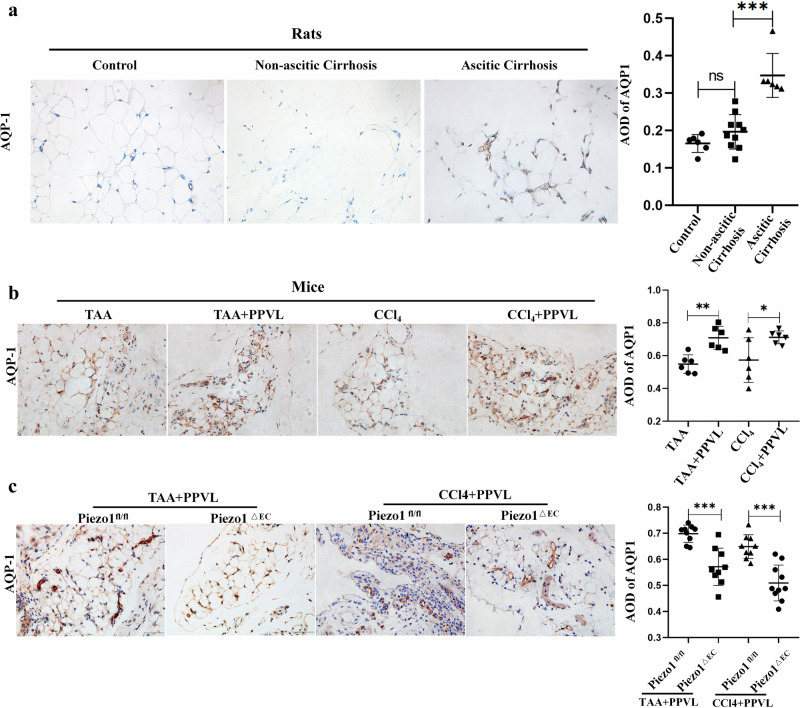
Portal hypertension induces AQP1 expression in peritoneal endothelium through Piezo1. **a** The expression of peritoneum AQP1 in cirrhotic rats with/without ascites. **b** AQP1 expression in the peritoneum collected from cirrhotic mice with or without PPVL. **c** AQP1 expression in the peritoneum collected from Piezo1^△EC^ mice or Piezo1^fl/fl^ mice treated with TAA/CCl_4_ plus PPVL. *P < 0.05, **P < 0.01, ***P < 0.001. ns, not significant.

**Fig. 6:**
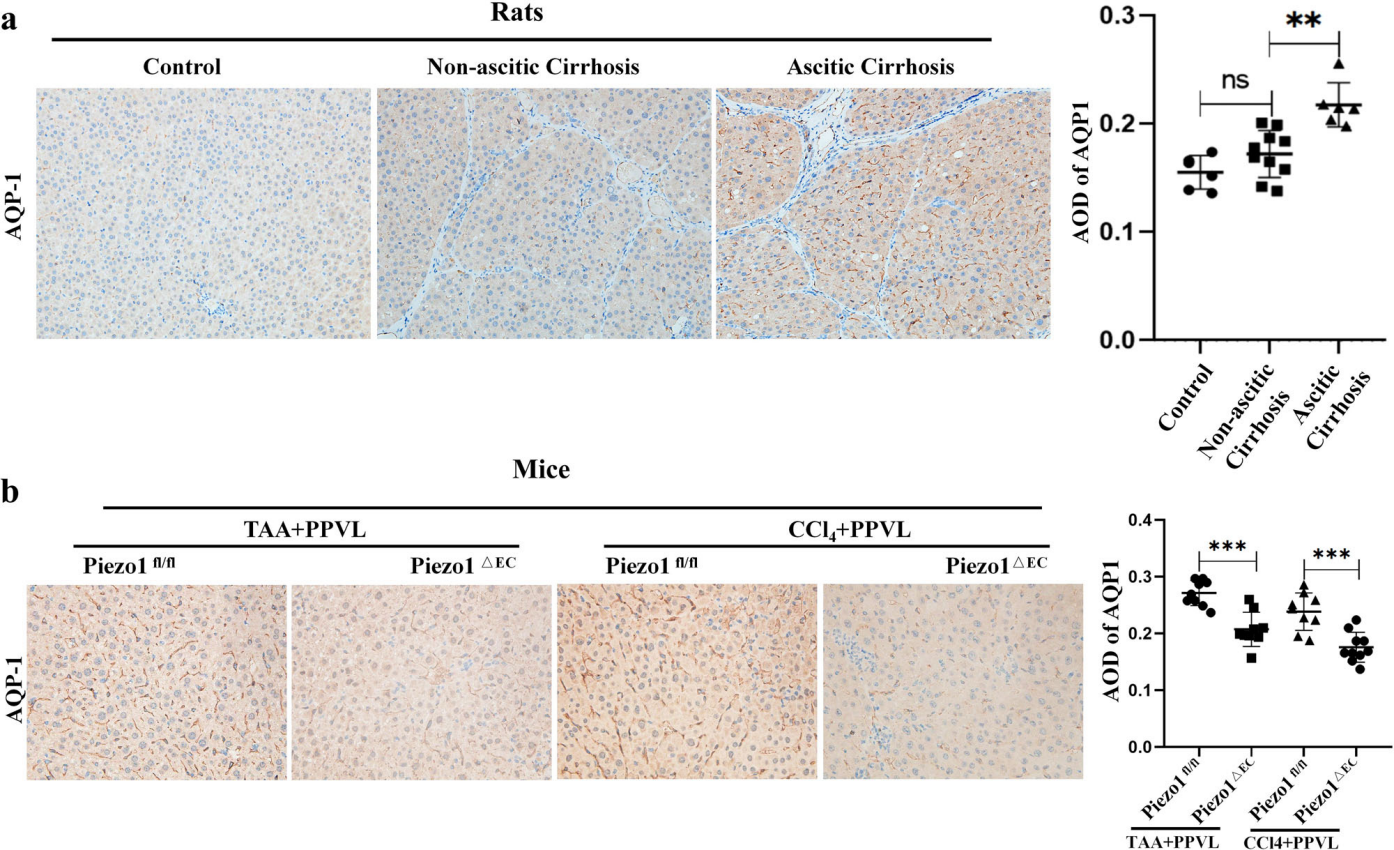
Hydrostatic pressure induces AQP1 expression in LSECs through Piezo1. **a** The expression of LSEC AQP1 in rats with/without ascites. **b** AQP1 expression in the LSECs collected from Piezo1^△EC^ mice or Piezo1^fl/fl^ mice treated with the TAA/CCl_4_ administration plus PPVL. AOD, average optical density. *P < 0.05, **P < 0.01, ***P < 0.001. ns, not significant.

To explore the mechanism underlying the contribution of Piezo1 to the development of cirrhotic ascites, we extracted and analyzed the expression profile database of HUVECs exposed to laminar shear stress in four GEO datasets. The results of differential expression analysis showed that aquaporin (AQP)1 mRNA increased significantly in HUVEC exposed to laminar shear stress (Fig. 4a–c). Single-cell sequencing also showed that high hydrostatic pressure increased AQP1 expression in the vascular endothelium of the lungs (Fig. 4d). AQP1 is the most abundant isoform of AQP in the peritoneum, and is very specifically located in capillary and venule endothelium^35–37^.More than 50% of the H_2_O transport in the peritoneum is mediated by AQP1 (ref. ^38^). In liver cirrhosis, peritoneal endothelial cells are subjected to mechanical pressure caused by portal hypertension^12^. The peritoneum contributed mainly to the formation of ascites in cirrhotic rats^34^. Thus, AQP1 expression in the peritoneum was determined and the results of immunostaining showed that AQP1 was mainly expressed in the peritoneal microvascular endothelium (Supplementary Fig. 7a). Interestingly, AQP1 expression in the peritoneum increased significantly in cirrhotic rats with ascites compared with cirrhotic rats without ascites (Fig. 5a). Increased expression of AQP1 in peritoneum was also observed in patients with cirrhosis with ascites (Supplementary Fig. 7b). Additionally, in murine models, AQP1 expression increased significantly in the TAA/CCl_4_ + PPVL group compared with the TAA/CCl_4_ group (Fig. 5b). This reveals that portal hypertension induced AQP1 expression in the peritoneum. Importantly, in cirrhotic mice with ascites, AQP1 expression in peritoneal endothelium decreased significantly in Piezo1^△EC^ mice compared with Piezo1^flox/flox^ mice (Fig. 5c). This indicates that portal hypertension induced AQP1 expression in peritoneal endothelium through Piezo1 in a murine model of liver cirrhosis. Ascites was also produced by the liver capsule in cirrhotic rats^34^. In liver cirrhosis, LSECs are subjected to mechanical stretch^12^. Thus, we hypothesized that LSECs are involved in ascites formation in liver cirrhosis. AQP1 expression in the liver was determined and the results of immunostaining showed that AQP1 was mainly expressed in the LESCs (Supplementary Fig. 8a, b). First, immunohistochemical staining of AQP1 in LESCs demonstrated AQP1 expression in patients with cirrhosis with ascites was higher than in patients without cirrhosis and patients with cirrhosis without ascites (Supplementary Fig. 8c). Second, AQP1 expression in LSECs increased significantly in cirrhotic rats with ascites compared with cirrhotic rats without ascites (Fig. 6a). These results indicate that portal hypertension induced AQP1 expression in LSECs. Third, in TAA/CCl_4_ + PPVL-treated mice, AQP1 expression in LSECs decreased significantly in Piezo1^△EC^ mice compared with Piezo1^flox/flox^ mice (Fig. 6b), indicating that portal hypertension induced AQP1 expression in LSECs through Piezo1. PPVL may reduce the shear forces on LSECs in a murine model of liver cirrhosis, and AQP1 expression in LSECs decreased in the CCL4 + PPVL group compared with CCl4-treated mice, but statistical analysis showed no significant difference between the two groups (Supplementary Fig. 8d).

**Fig. 7:**
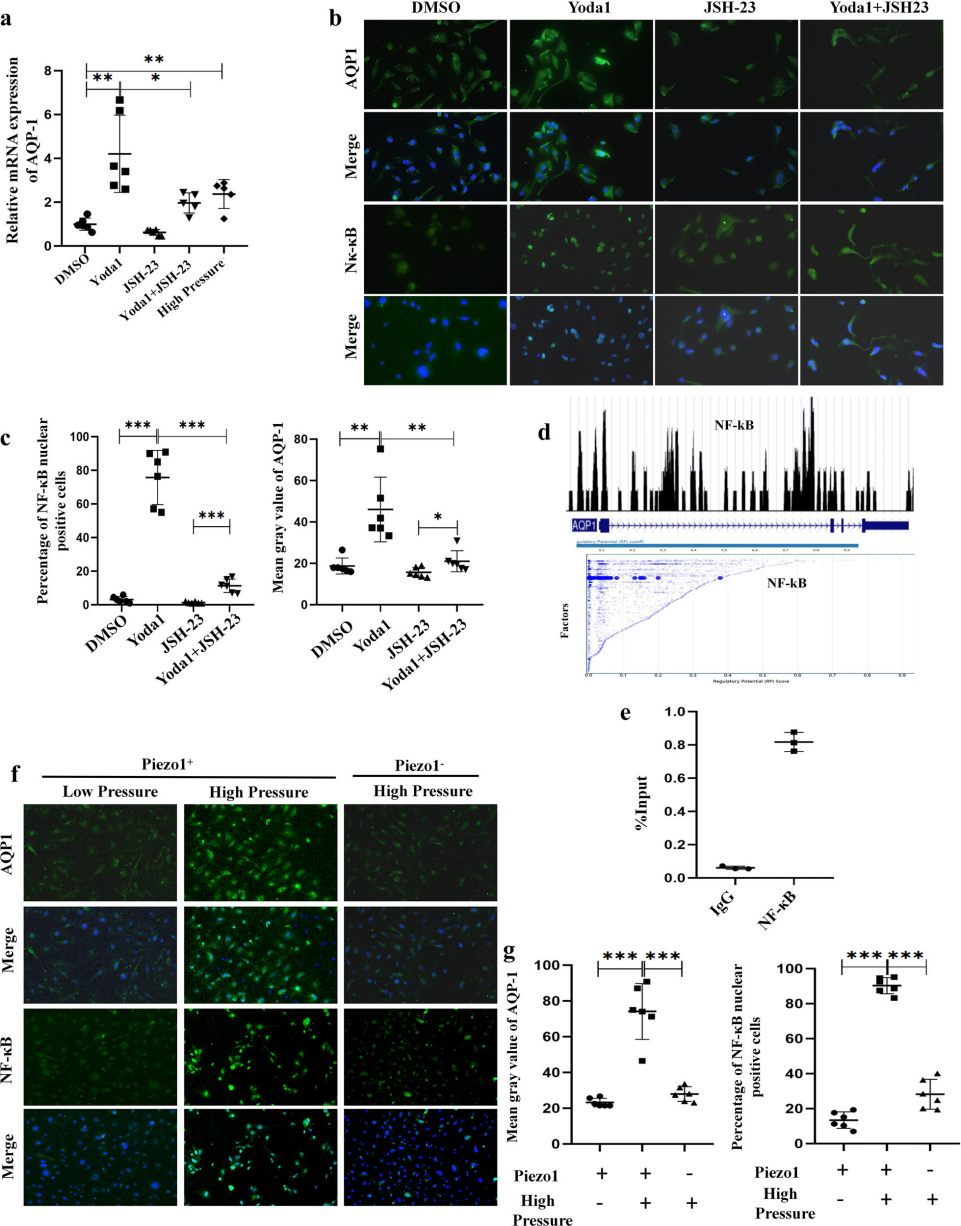
Piezo1 increased AQP1 expression in human primary LESCs through NF-κB. **a** The relative mRNA expression of AQP1 in primary LESCs treated with Yoda1 (a Piezo1 agonist), high pressure and JSH-23 (an inhibitor of NF-κB nuclear translocation). **b** The translocation of NF-κB and AQP1 expression in LESCs treated with Yoda1, a Piezo1 agonist and JSH-23, an inhibitor of NF-κB nuclear translocation. **c** Statistical data of translocation of NF-κB (left) and the protein expression of AQP1 (right) in LESCs exposed to Yoda1 and JSH-23. **d** Bioinformatics analysis showing the relationship between NF-κB and AQP1 using the Cistrome Data Browser (top) and NF-κB showing high regulatory potential to AQP1 using the Cistrome Data Browser (bottom). **e** The ChIP assay. LESCs were treated with 1% formaldehyde to crosslink chromatin and subjected to immunoprecipitation using anti-NF-κB, with preimmune IgG as a control. The precipitated chromatin fragments were further analyzed by RT−qPCR using the AQP1 primer. **f** The translocation of NF-κB and AQP1 expression in LESCs exposed to high hydrostatic pressure. **g** Semi-quantitative analysis of translocation of NF-κB (left) and AQP1 (right) in LESCs exposed to high pressure. *P < 0.05, **P < 0.01 and ***P < 0.001. ns, not significant.

**Fig. 8:**
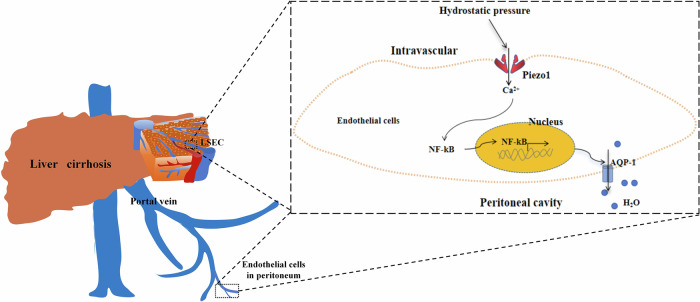
Graphical summary of the signaling pathway in which portal hypertension promotes ascites formation via the Piezo1−NF-κB−AQP1 pathway in liver cirrhosis. Portal hypertension results in the activation of Piezo1 in peritoneal endothelial cells/LSECs, which leads to the translation of NF-κB into the nucleus. NF-κB increases AQP1 expression and AQP1 promotes ascites formation through changing water transport across the peritoneal membrane/liver capsule.

The transcription factor nuclear factor kappa-B (NF-κB) has served as a standard for inducible transcription factors for decades and was reported to act downstream of Piezo1 (ref. ^39^). Thus, we determined whether endothelial Piezo1 regulated AQP1 expression via NF-κB. First, Piezo1 activation by Yoda1 induced the translocation of NF-κB and upregulated AQP1 expression in human primary LESCs in vitro (Fig. 7a–c). Then, AQP1 expression in LSECs was inhibited by JSH-23, an inhibitor of NF-κB nuclear translocation, which illustrated the involvement of NF-κB in Piezo1-induced upregulation of AQP1 expression. Next, we examined the possibility of NF-κB-binding sites in the AQP1 promoter region (Fig. 7d). ChIP−qPCR results showed that NF-κB could significantly bind to the AQP1 promoter (Fig. 7e). These results reveal that Piezo1 activation increased the expression of AQP1 through NF-κB. Importantly, Piezo1 activation in LSECs was induced when exposed to high hydrostatic pressure, which was similar to LSEC/endothelial dysfunction in cirrhosis, and subsequently triggered nuclear translation of NF-κB and stimulated AQP1 expression. In Piezo1-deficient LESCs, nuclear translation of NF-κB was blocked and AQP1 expression was suppressed (Fig. 7f, g). The role of NF-κB in endothelial Piezo1-regulated AQP1 expression was also investigated with HUVECs, and similar results were found (Supplementary Fig. 9). Immunofluorescence of the intracellular distribution of NF-κB also showed that high pressure induced NF-κB nuclear translocation in cirrhotic mice with ascites, and NF-κB nuclear translocation was inhibited in Piezo1^△EC^ mice (Supplementary Fig. 10). Together these results demonstrate that Piezo1 activation due to high hydrostatic pressure induced AQP1 expression via NF-κB (Fig. 8).

## Discussion

In our study, we demonstrated that portal hypertension plays a fundamental role in ascites formation and explored the underlying mechanism in liver cirrhosis. A systematic review and meta-analyses on the contribution of portal hypertension to ascites formation have been lacking; thus, a systematic review and meta-analysis was performed here. The results demonstrated that portal pressure increased significantly in patients with cirrhosis with ascites compared with cirrhotic patients without ascites, revealing the positive correlation between portal hypertension and ascites formation in patients with liver cirrhosis. Additionally, in cirrhotic rats with ascites, we found that portal pressure increased significantly in cirrhotic rats with ascites compared with rats without ascites. Together these results demonstrate the close relation between portal hypertension and ascites formation in liver cirrhosis.

The mechanism underlying the contribution of portal hypertension to ascites formation in liver cirrhosis is an important issue. A reliable and reproducible murine model is a prerequisite for unraveling this mechanism. Unfortunately, a reliable murine model for portal hypertensive ascites has not been available. Our study showed a murine mode with liver cirrhosis and ascites was not established successfully because cirrhotic mice induced by TAA/CCl_4_ had lower portal pressure compared with cirrhotic rats with ascites. In consideration of the critical contribution of portal hypertension to ascites formation, a novel murine model was established through TAA/CCl_4_ administration followed by PPVL. Portal pressure increased significantly in cirrhotic mice after PPVL. Within 1 week after PPVL, the accumulation of ascites was observed, and some mice produced visible ascites. This demonstrates the essential role of portal hypertension in the development of ascites resulting from liver cirrhosis. Importantly, the amount of ascitic fluid produced by CCl_4_ + PPVL-treated mice was four times more than that of a previous model^40^. This represents a novel and effective method to produce a murine model of liver cirrhosis and ascites for investigating the pathogenesis and therapy of ascites in cirrhosis.

Does portal hypertension alone result in a large accumulation of ascitic fluid? A murine model of non-cirrhotic portal hypertension was established through PPVL. When portal vein in mice were cannulated with a 27-gauge needle, similar levels of portal pressure were observed between TAA/CCl_4_ + PPVL-treated mice and PPVL-treated mice. Interestingly, the accumulation of ascites was not observed in PPVL-treated mice (Supplementary Fig. 2). Thus, presinusoidal portal hypertension does not result in the formation of ascites. In liver cirrhosis, ascites was mainly produced by the hepatic capsule and peritoneum^34^. In liver cirrhosis, increased AQP1 expression was observed in LSECs or peritoneal endothelium^41^. Previous studies have shown that the enhanced expression of AQP1 in LSEC was involved in pathological angiogenesis^42^, and our study suggested that AQP1 in LSECs and peritoneal endothelial cells contributed to ascites formation. Additionally, our results revealed that endothelial cells participated in the formation of cirrhotic ascites through the Piezol−NF-ĸB−AQP1 pathway in liver cirrhosis.

PPVL-treated rats did not develop ascites^43^, thus portal hypertension was not the only determinator in the development of cirrhosis-related ascites. In liver cirrhosis, sodium and water retention, hypoproteinemia, systemic inflammation and severe liver injury exert synergistic effects on ascites formation^3,44,45^ The underlying mechanism involved in portal hypertensive ascites was explored in our study. Piezo1, a 286-kDa transmembrane cation channel, detects the changes in membrane tension and membrane curvature, then transduces mechanical signals into biological signals by the influx of cations and downstream signaling pathways. Our study demonstrated that portal hypertension induces Piezo1 activation in peritoneal endothelial cells/LSECs, which gives rise to the accumulation of ascites. In pulmonary edema, Piezo1 in endothelial cell mediates pressure-induced lung vascular hyperpermeability via disruption of adhere junctions^11^. Aquaporin water channels are the regulators of transcellular water flow. Water molecules in peritoneal vessels are mainly transported by aquaporins under hydrostatic pressure or osmotic pressure^46,47^, and AQP1 is the most abundant isoform in the peritoneum, mainly located on peritoneal microvessels and venular endothelium^38^. Several studies have described hepatic AQP1 expression and AQP1 urinary excretion in liver cirrhosis^42,48,49^. Our study revealed that portal hypertension induced AQP1 expression in peritoneal endothelial cells. Endothelial cell-specific AQP1 knockout mice confirmed the crucial role of endothelial AQP1 in peritoneal fluid transport^37^. AQP1 overexpression is involved in ascites formation though changing water transport across the peritoneal membrane^38,50,51^. In Piezo1^△EC^ mice, increased AQP1 expression was not observed in cirrhotic mice, which indicated Piezo1 contributed to the development of portal hypertensive ascites via AQP1. Similar results were also observed in LSECs in a murine model of liver cirrhosis. In addition, our study demonstrated that Piezo1 activation induced AQP1 expression via NF-ĸB through an in vitro hydrostatic pressure model.

Lymphatic vessels are crucial for maintaining abdominal fluid homeostasis^52^. Mesenteric lymphatic vessels are dysfunctional in experimental liver cirrhosis, which contributes to the formation of ascites resulting from portal hypertension^53,54^. Thus, the lymphatic vasculature was additionally evaluated in this study. A decrease in smooth muscle cells coverage surrounding the lymphatic vessels was observed in cirrhotic mice with ascites compared with cirrhotic mice without ascites, indicating lymphatic abnormality in cirrhotic mice with ascites (Supplementary Fig. 11a). Additionally, in murine models of liver cirrhosis and ascites, the smooth muscle cells lymphatic coverage increased in Piezo1^△EC^ mice compared with Piezo1^flox/flox^ mice (Supplementary Fig. 11b). This indicates that Piezo1 in the endothelial cells of lymphatic vessels is involved in the formation of portal hypertensive ascites.

There were also some limitations in this study. First, we did not create murine models of liver cirrhosis and ascites through bile-duct ligation plus PPVL due to high mortality. Second, previous studies have demonstrated AQP1 overexpression in peritoneal endothelial cells increases water permeability across the peritoneal membrane^37,45^. However, the crucial role of endothelial AQP1 in portal hypertensive ascites should be investigated using endothelial cell-specific AQP1 knockout mice. This study will be performed to investigate the role of endothelial AQP1 in the formation of portal hypertensive ascites and the underlying mechanism in the future.

In summary, in liver cirrhosis, portal hypertension is critical to the development of ascites. A meta-analysis and animal models revealed the essential role of portal hypertension in the development of ascites caused by liver cirrhosis. Piezo1 in the peritoneal endothelium/LSECs contributes to the formation of portal hypertensive ascites via the NF-ĸB−AQP1 pathway in liver cirrhosis. These results reveal that inhibition of the Piezo1−NF-ĸB−AQP1 pathway may be a useful therapeutic strategy for reducing ascites formation in liver cirrhosis.

## Summary

### What Is Current Knowledge

- In liver cirrhosis, portal hypertension is critical to the development of ascites.
- The mechanically activated ion channel Piezo1 is initiated by elevated pressure and other mechanical stimuli.
- AQP1 is a water channel that facilitates water transport across cell membranes.

### What Is New Here

- A meta-analysis and rat model demonstrated a positive correlation between portal hypertension and ascites formation in liver cirrhosis.
- A novel murine model of liver cirrhosis revealed the essential role of portal hypertension in the development of cirrhotic ascites.
- Piezo1 in endothelial cells contributed to the development of portal hypertensive ascites in liver cirrhosis via the nuclear factor kappa-B−aquaporin1 pathway.

### Implications for Patient Care

- A novel murine model represents a reliable and effective model for investigating the pathogenesis and therapy of ascites in liver cirrhosis. Portal hypertension contributes to ascites formation via the Piezo1−nuclear factor kappa-B−aquaporin1 pathway, which provides a therapeutic candidate for the management of ascites in cirrhosis in the future.

## Supplementary information

Supplementary Information

## Data Availability

All data generated in this study are available from corresponding author on reasonable request.
