# Supplementary material for: Portal hypertension contributes to ascites formation via the Piezo1−nuclear factor kappa-B−aquaporin1 pathway in liver cirrhosis
Source: Exp Mol Med. 2025 Oct 1;57(10):2305–16. doi: 10.1038/s12276-025-01554-6 (PMC12586457; doi:10.1038/s12276-025-01554-6)
Supplement: Supplementary file 1 — Supplementary Information [file 12276_2025_1554_MOESM1_ESM.pdf]

Supplementary data

Portal hypertension contributes to ascites formation via piezo1-nuclear factor kappa B-aquaporin1 pathway in liver cirrhosis

Ning Wei[ab], Li Du[a], Zhuanglong Xiao[a], Lei Zhang[a], Yangyang Zhou[a], Haonan Gao[a], Minghui Liu[a], Chengbo Wang[a], Xiaohua Hou[a], Yan Li[c], Yuhu Song[a]

[a] Department of Gastroenterology, Union Hospital, Tongji Medical College, Huazhong University of Science and Technology, Wuhan 430022, China.

[b] Department of Gastroenterology, The Central Hospital of Wuhan, Tongji Medical College, Huazhong University of Science and Technology, Wuhan, China.

[c]Department of Gastroenterology, the First People's Hospital of Yunnan Province, Affiliated Hospital of Kunming University of Science and Technology

The type of manuscript: original Research

Ning Wei and Li Du contributed equally to this study.

Corresponding author:

Yuhu Song

Department of Gastroenterology, Union Hospital, Tongji Medical College, Huazhong University of Science and Technology; Wuhan 430022, China; Email: yuhusong@163.com; Tel: 86-15007187581

Yan Li

Department of Gastroenterology, the First People's Hospital of Yunnan Province, Affiliated Hospital of Kunming University of Science and Technology; Kunming 650032, China; Email: 9y140172@kust.edu.cn; Tel: 86-13808793358

Running title: Piezo1 in portal hypertensive ascites

Financial support: This work was supported by National Natural Science Foundation of China (No. 82470662, 82070631) and Yunnan revitalization talent support program (XDYC-YLWS-2023-0066).

Potential competing interests: The authors have no conflict of interest.

Material and methods

Meta analysis

Search strategy

PubMed, EMBASE, Web of Science and Cochrane Library were searched by two authors independently to identify relevant studies on the measurement of portal pressure in cirrhotic patients with or without ascites. Search strategy was based on the following terms and keywords: "portal hypertension" OR "portal pressure" OR "hepatic venous pressure gradient (HVPG)" AND "ascites" AND "cirrhosis" AND "patient".

Inclusion and exclusion criteria

Two authors independently screened articles meeting the following inclusion criteria: (1) cirrhotic patients with or without ascites who received hepatic venous pressure gradient (HVPG) measurements;(2) eligible studies should report the HVPG values expressed as mean and standard deviation (SD). Exclusion criteria were as follows: (1) patients who used selective beta-blockers (NSBBs); (2) patients who received radiologic intervention [transjugular intrahepatic portosystemic shunt (TIPS) or balloon-occluded retrograde transvenous obliteration (BRTO)]; (3) patients who

received endoscopic therapy [endoscopic variceal ligation (EVL), endoscopic cyanoacrylate glue injection (ECGI)]; (4) patients who received surgery (surgical portosystemic shunts, devascularization); (5) duplicate publications; (6) case reports, reviews or meta-analyses, guidelines, seminar reports.

Data extraction and data analysis

Two authors extracted the data independently, and a third investigator resolved the inconsistency. Data including first author, publication year, country, etiology of cirrhotic, sample size, HVPG, were extracted (Supplementary Table 1). Then the study quality was evaluated. Cohort or case–control studies were evaluated by the methodological index for non-randomized studies (minors)[1] (Supplementary Table 2).

Statistical analysis

All meta-analyses were conducted by using STATA version 12.0 (Stata Corp, College Station, Texas, USA). HVPG was pooled using either random-effects model or fixed-effects model according to the result of heterogeneity analysis which was tested by Q and I^2 statistics, and $p<0.05$ or $I^2>50\%$ indicated significant heterogeneity. Firstly, we evaluated the difference in HVPG between cirrhotic patients with ascites and cirrhotic patients without ascites. Then, we pooled the absolute HVPG values of cirrhotic patients (cirrhotic patients without ascites, cirrhotic patients with ascites, and cirrhotic patients with refractory ascites) .

Animals

Sprague-Dawley (SD) rats were purchased from Beijing Vital River Laboratory Animal Technology Co. Ltd. (Beijing, China). C57BL/6 mice were purchased from Hubei Biont

Bio-technology Co., Ltd (Wuhan, China). Piezo1$^{flox/flox}$ mice (Cat. NO: NM-CKO-200275) were purchased from Shanghai Model Organisms Center, Inc and Cdh5-CreERT2 mice (Strain NO:T014691) were purchased from GemPharmatech Co., Ltd. (Nanjing, China). Piezo1$^{flox/flox}$ mice were crossed with Cdh5-CreERT2 mice[2] to generate endothelium-specific Piezo1-deficient mice (Piezo1$^{\triangle EC}$) with the deletion of exon 4 and 5 of Piezo1 cDNA (NCBI ID: 234839) after the injection of tamoxifen.

All animals were housed in specific pathogen-free (SPF) animal facility. Animal experiments were approved by the institutional animal care and use committee. The breeding environment was a constant temperature and humidity barrier system, and the day and night light and dark alternate time was 12h/12h.

Isolation of Liver sinusoidal endothelial cells (LSECs) and peritoneal vascular endothelial cells (PVECs)

Mouse LSECs were obtained by in situ perfusion with collagenase type IV and DNAase followed by differential centrifugation[3]. The peritoneal tissues were cut into small pieces with the scissors, and the tissue mixtures were digested for approximately 1 hour. Then, LSECs or PVECs were isolated by magnetic cell separation (MACS) using anti-mouse CD146 or CD31 magnetic beads (Miltenyi, Inc.), respectively. The purity of endothelial cells was assessed by flow cytometry staining using CD31 antibody.

Patients' peritoneum and liver specimens

The paraffin sections of patient's peritoneal and liver samples were obtained from our hospital. The related research protocol was approved by the Ethics Committee of Union Hospital, Tongji Medical School, Huazhong University of Science and Technology

(ethics approval No. [2021] 0003-04).

Animal models of liver cirrhosis

Rat model of liver cirrhosis was established with subcutaneous injection of carbon tetrachloride (CCl_4). A novel murine model of liver fibrosis with ascites were established through the administration of chemicals [thioacetamide (TAA) or CCl_4] plus partial portal vein ligation (PPVL).

Carbon tetrachloride (CCl_4)-induced rat model of liver cirrhosis

Male rats (10 weeks old) were injected with 40% carbon tetrachloride diluted in olive oil subcutaneously for 17 weeks after 1 week of receiving phenobarbital (0.3g/L in drinking water).

TAA-induced murine model of liver cirrhosis with ascites

TAA-induced murine model of liver cirrhosis with ascites was established through the administration of TAA plus PPVL. In brief, male mice (8-10 weeks old) were intraperitoneally injected with 150 mg/kg TAA three times a week for 8 weeks. Then, the mice received partial portal vein ligation (PPVL) at 7th week after TAA administration.

In the surgical procedure to increase portal hypertension by PPVL, the mice were anaesthetized, and a midline abdominal incision was performed and the portal vein was separated from the surrounding tissue. The isolated portal vein was ligated with the 27G blunt-tipped syringe needle and 7-0 nylon sutures, and then the syringe was removed. Subsequent removal of the needle yielded a calibrated stenosis of the portal vein. For Piezo1$^{\triangle EC}$ and Piezo1$^{flox/flox}$ group, the mice received intraperitoneal injection

of tamoxifen (1mg /day) for 6 consecutive days at 3th week after TAA administration.

CCl_4-induced murine model of liver cirrhosis

Male mice (8-10 weeks old) were injected subcutaneously with CCl_4 diluted 5:5 (v/v) ratio in olive oil at a dose of 3 ml/kg twice a week for 12 weeks. For the CCl_4+PPVL model, mice would be performed PPVL at 11th week after CCl_4 administration. The specific surgical method was the same as above. For Piezo1$^{\triangle EC}$ and Piezo1$^{flox/flox}$ group, the mice received intraperitoneal injection of tamoxifen (1mg /day) for 6 consecutive days at 7th week after CCl_4 administration.

Ethics statement

All experiments involving animals were conducted according to the ethical policies and procedures approved by the ethics committee of the Institutional Animal Care and Use Committee (IACUC) of our institution (approval number: 3372)

Evaluation of ascites volume

At sacrifice, four strips of absorbing paper were placed in the abdominal cavity and removed after 3 min. Ascites amount was calculated by weight difference of strips before and after their placement[4,5].

Portal pressure measurement

Water column method was used to measure the portal pressure. Infusion needles connected to the glass-tube manometer were inserted into the portal vein. When the water column in the glass-tube manometer was stationary, the scale shown in the glass tube was the value of portal vein pressure[6].

Histology

Liver samples were fixed in 10% phosphate-buffered formalin, and paraffin-embedded specimens were cut into 5-μm-thick sections and stained with hematoxylin-eosin (H&E). For the assessment of collagen deposition, Sirius Red staining was performed using the staining assay kit.

Immunohistochemical staining

Formalin-fixed, paraffin-embedded tissue specimens were cut into 5-μm-thick sections. The slides were incubated with primary antibodies at 4 °C overnight, and then incubated with secondary antibodies at room temperature for 30 min. Staining process was performed using two step immunohistochemistry test kit (BOSTER Biological Technology). 3,3′-diaminobenzidine was used for detection.

Rivalta Test

Firstly, one drop (20 μL) of acetic acid was mixed with 8 ml of distilled water in a transparent tube. Then, 1 drop of the ascites was carefully dropped into the test solution. The Rivalta's test would be considered as positive when the drop retains its shape or slowly floats down to the bottom of the tube. If the drop disappears and the solution remains clear, the Rivalta's test would be defined as negative.

Serum liver function assay

Serum alanine aminotransferase (ALT), aspartate aminotransferase (AST), albumin (ALB) were measured using commercial kits purchased from the Nanjing Jiancheng Bioengineering Institute (Nanjing, China).

Cells

Human umbilical vein endothelial cells (HUVECs) were purchased from Thermo

Fisher Scientific(C0035C). The cell was maintained in DMEM medium supplemented with 10% fetal bovine serum (FBS), penicillin, and streptomycin in a humidified atmosphere containing 5% CO_2 at 37℃.

Immunofluorescent staining

HUVECs/peritoneum were fixed in a 4% paraformaldehyde fixative solution. The HUVECs/peritoneum were incubated with primary antibodies overnight at 4℃, followed by the incubation with secondary antibodies for 1 hour at room temperature. The slides were mounted 4',6-diamidino-2-phenylindole (DAPI), and the images were collected using confocal microscope. Antibodies used were listed in Supplementary Table 4.

Calcium imaging.

HUVECs were incubated with 1.0-μM Fluo-3 AM solution at 37°C for 15 min, then washed HUVEC using PBS, and the images were collected using confocal microscope.

Exposure of endothelial cells to hydrostatic pressure in vitro

To simulate the pathological state of endothelial cells in cirrhotic patients with portal hypertension, a novel in vitro pressure model was created through the exposure of endothelial cells to hydrostatic pressure. As shown in Figure. S4A, a layer of endothelial cells were planted on a trans-well with a diameter of 24 mm and a pore size of 0.4 μm, and a glass coverslip with a diameter of 2.4 cm was added on the HUVECs, and then a 100g weight was placed on the coverslip. The above device was placed in an incubator and the effect of hydrostatic pressure on endothelial cells was elevated after 48 h of intervention.

RNA isolation and RT-qPCR

Total RNA was isolated from HUVECs/LSECs using Trizol reagent, and 10 μg of total RNA was used for cDNA synthesis using an RT kit. Quantitative real-time reverse transcription (RT-qPCR) was carried out in LightCycler480 using SYBR Green Transcription Master (Roche Diagnostics, IN, USA). Transcripts were amplified using specific primer pairs， while GAPDH was used as internal control and quantified using the $2^{-\Delta\Delta Ct}$ method. Primers were listed as Supplementary Table 3.

Preparation of lentivirus expressing siRNA against Piezo1

The recombinant lentivirus expressing siRNA against Piezo1 and the control were packaged and purified by GeneChem Co, Ltd, (Shanghai, China). In brief, the oligo pairs of siRNAs against Piezo1were inserted into lentiviral expression vector GV248. Then, 293T cells were transfected with the Lentiviral expression vector carrying siRNA against Piezo1and packaging vectors (pHelper 1.0 and pHelper 2.0). The lentivirus carrying siRNA against Piezo1was produced according to the instruction of manufacture. The siRNA oligo pairs were used： PIEZO1-RNAi (27901-1)-a (5’-3’): Ccggga AGA CCA CAT TCA GGT GGA ACT CGA GTT CCA CCT GAA TGT GGT CTT CTT TTT g; PIEZO1-RNAi (27901-1)-b(5’-3’) : aattc aaaaa ga AGA CCA CAT TCA GGT GGA ACT CGA GTT CCA CCT GAA TGT GGT CTT C;

Construction of Piezo1-deificent HUVECs/LSECs

HUVECs/LSECs were infected with lentivirus expressing siRNA against Piezo1and the control. 72 hours after infection, cells were selected for 1-2 weeks using puromycin. Then, the selected cells were cultured with puromycin (5 μg/ml).

Analyzing data from GEO

To search differential expressed genes (DEGs) of HUVECs exposed to mechanical force, gene-expression profiles of HUVECs exposed to mechanical force were downloaded from the GEO (http://www.ncbi.nlm.nih.gov/geo) database. The GEO (http://www.ncbi.nlm.nih.gov/geo) database is a public database containing a large number of high-throughput gene expression and other functional genomics data sets. 4 datasets (GSE71164, GSE213099, GSE87534, GSE167024) were screened. GEO2R (http://www.ncbi.nlm.nih.gov/geo/geo2r/) was used to screen differential expressed genes between HUVECs and HUVECs exposed to laminar flow shear stress. Significant differential expressed genes (DEGs) were identified as those with a false discovery rate (FDR) value above the threshold (Q < 0.05) and fold-change >2.

Volcano plot was generated using the ggplot2 package to graphically reveal differential expressed genes between HUVECs and HUVECs exposed to laminar flow shear stress. GSE154959 was screened to figure out the influence of high pressure stimulation on endothelium of the lungs.

Chromatin immunoprecipitation (ChIP) assay

Briefly, cells were cross-linked by 1% formaldehyde. Chromosome DNA was extracted according to the manufacturer’s instructions provided by SimpleChIP® Plus Enzymatic Chromatin IP Kit (Magnetic Beads) (Cell Signaling Technology, Cat#9005) and

precipitated by using specific anti-NF-κB antibody. Rabbit IgG was used as negative control. Finally, DNA was purified and quantified by qPCR.

Statistical analysis

Continuous variables were expressed as mean ± SEM and analyzed with student's test or Mann-Whitney's U test. A $P<0.05$ was considered as significant. Statistical analyses were performed using SPSS version 26.0 (SPSS Inc., Chicago, Illinois, USA).

Supplementary Table 1 Basic information for the literature included in the meta-analysis

First Author	Years	Country	Etiology of cirrhosis	Number of patients with/without ascites	Average HVPG of patients with ascites (mmHg)	Standard deviation of HVPG in patients with ascites(mmHg)	Average HVPG of patients without ascites(mmHg)	standard deviation of HVPG in patients without ascites (mmHg)
Annalisa Berzigotti	2011	Spain	Virus:109; alcohol:35; cryptogenic: 8; other: 9	0/161			12	4.3
Ankur Jindal	2022	India	Alcohol: 8;hepatitis B: 17;hepatitis C：28；NASH 49; cryptogenic 17; others 36	0/132			6.87	1.34
Diego Rincon	2014	Spain	Alcohol:7; hepatitis C:2; alcohol& hepatitis C :2； NASH: 1	12/0	21.8	6.2		
Luis Ruiz Del Arbol [&]	1997	Spain	Alcohol:24; hepatitis C:12; cryptogenic:1	10/0	19.5	1.5		
				27/0	17.1	4.2		
Angelo Luca[&]	1995	Spain	Alcohol:7; hepatitis C: 3; hepatitis B:4; Cryptogenic: 4	9/0	20.8	2.6		
				9/0	19.7	4.1		
Richard Morea[&]	1987	France	Alcohol:12	6/0	20.3	2.3		
				6/0	20.5	8.2		
Antoine Hadengue	1990	France	Alcohol 12	12/0	16.8	4.8		
Angelo Luca	1994	Spain	Alcohol: 5; HCV:3; HBV:1; cryptogenic:1	10/0	20	3.8		

Virginia Hern á ndez Gea[&]	2012	Spain	Alcohol:14; HCV:48; Alcohol& HCV:11	0/40			17.4	3
				0/38			16.8	3
M. Wadhawan	2006	India	Viral:104; alcohol: 40; cryptogenic: 26; miscellaneous: 6	80/96	18.5	5.6	16.6	7.6
Ghulam Mohamad Gulzar	2009	India	-	20/20	15.7	2.4	13.9	3.1
Yoshiyuki Narahara	2009	Japan	Alcohol: 9; hepatitis C: 12; hepatitis B:3; others: 4	18/10	21.2	5.6	16.3	3.1
Laura Turco*	2017	Italy	Viral: 86; alcohol:34; alcohol +viral: 21; NASH:18; miscellaneous: 18	87/90	19.55	0.83	16.77	0.4
			refractory ascites	39/0	21.52	0.74		
Isabelle Colle*	2000	France	Alcohol 74; alcohol&hepatitis B virus: 6; alcohol&hepatitis C virus: 2; hepatitis B virus 8; hepatitis C virus: 5; hepatitis B&C virus: 1; hepatitis B, C and D virus: 1； auto-immune hepatitis: 1; Primary biliary cirrhosis:3; haemochromatosis 3; undetermined 14;	92/29	18.5	0.8	15.8	0.7
			refractory ascites	47/0	20.1	0.8		
Yingying YANG	2003	Taiwan,China	Hepatitis B and/or hepatitis C 52; alcohol: 8；Primary biliary cirrhosis:1; Hemochromatosis:1；cryptogenic Cirrhosis: 5	33/34	18.4	1.1	15.1	0.2

Wui Chiang Lee	1999	Taiwan,China	Viral hepatitis-related (either hepatitis B or C): 138；alcohol :41; primary biliary cirrhosis :2;haemochromatosis:2; cryptogenic: 2 ；	28/64	13.7	0.6	16.4	1.3
R. Moreau	1993	France	alcoholic cirrhosis 91	63/28	19.1	0.7	15.8	0.8
Hong Chiang Meng	1994	Taiwan, China	HBV and/or HCV:149; alcohol:13; primary biliary cirrhosis:1; Cryptogenic: 30	73/120	17.8	4.7	14.9	5.1

*the patients with refractory ascites were enrolled in the study; &two cohort in one study, HVPG data of each cohort were provided when meta-analysis was performed

Supplementary Table 2 The results of methodological index for non-randomized studies (MINORS) for the included literature in the meta-analysis.

MINORS	Box1	Box2	Box3	Box4	Box5	Box6	Box7	Box8	Box9	Box10	Box11	Box12	Score
Annalisa Berzigotti/2011	2	2	2	2	2	2	2	0	_	_	_	_	14/16
Ankur Jindal/2022	2	2	2	2	2	2	2	0	_	_	_	_	14/16
Diego Rincon/2014	2	0	2	2	2	2	0	0	_	_	_	_	10/16
Luis Ruiz Del Arbol/1997	2	0	2	2	2	2	2	0	_	_	_	_	12/16
Angelo Luca/1995	2	0	2	2	2	2	2	0	_	_	_	_	12/16
Richard Morea/1987	2	0	2	2	2	2	2	0	_	_	_	_	12/16
Antoine Hadengue/1990	2	0	2	2	2	2	2	0	_	_	_	_	12/16
Angelo Luca/1994	2	0	2	2	2	2	2	0	_	_	_	_	12/16
Virginia Hern á ndez Gea/2012	2	2	2	2	2	2	2	0	_	_	_	_	14/16
M. Wadhawan/2006	2	2	2	2	2	2	2	0	2	2	0	2	20/24
Ghulam Mohamad Gulzar/2009	2	2	2	2	2	2	2	0	2	2	0	2	20/24
Yoshiyuki Narahara/2009	2	0	2	2	2	2	2	0	2	2	0	2	18/24
Laura Turco/2017	2	2	2	2	2	2	2	0	2	2	0	2	20/24
Isabelle Colle/2000	2	0	2	2	2	2	2	0	2	2	0	2	18/24
Yingying YANG/2003	2	0	2	2	2	2	2	0	2	2	0	2	18/24
Wui Chiang Lee/1999	2	0	2	2	2	2	2	0	2	2	0	2	18/24
R. Moreau/1993	2	0	2	2	2	2	2	0	2	2	0	2	18/24
Hong Chiang Meng/1994	2	0	2	2	2	2	2	0	2	2	0	2	18/24

Box1: specific research purpose; box2: the consistency of participants; box3: Collection of the expected data; box4: The endpoint index can accurately reflect the research purpose; box5: Objectivity of the evaluation of the endpoint indicators; box6: sufficient follow-up time; box7: loss to follow-up% ≤ 5%; box8: estimated sample size. box 9:an adequate control group. box10: contemporary groups. box11: baseline equivalence of groups. box12: Adequate statistical analyses. 0: Not reported; 1: Reported with inadequate information; 2: Reported and provided adequate information; the global ideal score being 16 for non-comparative studies and 24 for comparative studies.

Supplementary Table 3 Primer sequences of qPCR used in our study

Gene	Forward primer (5′- 3′)	Reverse primer (5′- 3′)
Human		
Gαq/11(NM_002072)	CACGACGCTCCACATGAC	CAGCCAGGTCCAGATACAC
Piezo1 （NM_001142864）	CATCTTGGTGGTCTCCTCTGTCT	CTGGCATCCACATCCCTCTCATC
Trpv4(NM_021625)	CTACGGCACCTATCGTCACC	TTAGGCGTTTTCTTGTGGGTCA
Trpv1(NM_080706)	AGTATTTCCTGCAGAGGCGG	CAAGGCCAGGGAGAATACC
Gpr68(NM_001177676)	GTTTGAAGGCGGCAGAAATG	GTGGAATGAGGAGGCATGAA
Aqp1(NM_001185060)	ATTTTCTGGGTGGGGCCATT	GGGCCAGACCCCTTCTATTT
GAPDH(NM_001256799)	GCAGGGGGGAGCCAAAAGGGT	TGGGTGGCAGTGATGGCATGG
AQP1(for Chip-qPCR)	CAAGTGTGTGAGCAGGACTC	TTCTCTTGATTCCTAGAGGTGGT
Mouse		
Piezo1(NM_001037298)	TCTACTGGCTGTTGCTGCC	TTCAGGTCCAGCCTCGTAAC
GAPDH(NM_008084)	TGAAGGTCGGTGTGAACGG	GTGAGTGGAGTCATACTGGAA

Note: Gαq/11, G protein subunit alpha q11; Gpr68, G protein-coupled receptor 68; TRPV1, transient receptor potential cation channel subfamily V member

1; TRPV4, transient receptor potential cation channel subfamily V member 4; AQP1, aquaporin1.

Supplementary Table 4 Key resources table

Reagents or resources	Source	Application	Catalogue number
AQP-1	Affinity	IHC/IF	AF5231
Fluo-3AM	Beyotime	Calcium indicators	S1056
CD31	R&D	IF	AF3628-SP
NF-κB	CST	Chip	8242T
CD34	Servicebio	mIHC	GB11063
Stabilin 2	Affinity	IF	DF14269
Podoplanin	ABclonal	IF	A21748
alpha SMA	Affinity	IF	BF9212
NF-κB	Abcam	IF	Ab32536
**Chemicals, Peptides, and Recombinant Proteins**			
Alanine aminotransferase Assay Kit	Nanjing Jiancheng Bioengineering Institute	Serum ALT concentration testing	C009-2-1
Aspartate aminotransferase Assay Kit	Nanjing Jiancheng Bioengineering Institute	Serum AST concentration testing	C010-2-1
Albumin assay kit	Nan jing Jiancheng Bioengineering Institute	Serum ALB concentration testing	A028-2-1
Sirius red staining	Biossci	Sirius red staining	BP094
DNA marker	Vazyme	Genotyping	MD104-02-AA
Mouse Direct PCR Kit	Bimake	Genotyping	B40013
Triton® X-100	Biofroxx	IF	1139ML100
HY Nucleic acid dyes	HYCEZMBIO	Genotyping	HY70303-0.5ML
DAPI	AntGene	IF	ANT046
Thioacetamide	TCI	Murine model of liver	T0187

(TAA)		fibrosis	
Carbon tetrachloride	Makclin	Murine model of liver fibrosis	C822982
Olive oil	Makclin	Solvent for CCl4	O815211
Trizol reagent	Invitrogen	RNA extraction	15596-018
2× ChamQ SYBR qPCR Master Mix[a]	Vazyme	RT-PCR	Q311-02-AA
5*HiScript III RT SuperMix[a]	Vazyme	RT	R323-01
LV-PIEZO1-RNAi	Shanghai Genechem Co., LTD.	Lentivirus	27901-1
Tamoxifen	Sigma-Aldrich	Gene knockout	T5648-1G
Hematoxylin	BaSO	Tissue staining	BA4041
Eosin Y	SIGMA	Tissue staining	230251
HLSECs	Pricella	Primary cells	CP-H047
HUVECs	Thermo Fisher Scientific	Cell line	C0035C
CD31 MicroBeads mouse	Miltenyi	Magnetic cell sorting	130-097-418
CD146 (LSEC) MicroBeads mouse	Miltenyi	Magnetic cell sorting	130-092-007
LS Separation columns	Miltenyi	Magnetic cell sorting	130-042-401
MACS MultiStand	Miltenyi	Magnetic cell sorting	130-042-303
SimpleChIP kit	CST	Chip-qPCR	9005
MightyAmp™ for Real Time	Takara	Chip-qPCR	R075A
CD31	R&D	Flow cytometry	AF3628-SP

Software and Algorithms

Adobe Photoshop CS6	Adobe	Photo	Version 19.1.2
Stata software	StataCorp LLC	Photo	Version 15.0
GraphPad Prism	GraphPad Software	Photo	Version 5.0.1
SPSS	SPSS Inc.	Statistical analysis	version 22

Results:

Supplementary Table 5 Mortality data of animal models.

Models	Mortality (%)
Rat model of liver cirrhosis	11.11
C57BL/6 murine model of TAA induced liver cirrhosis	7.69
C57BL/6 murine model of CCl_4 induced liver cirrhosis	7.14
C57BL/6 murine model of PPVL	7.14
C57BL/6 murine model of TAA plus PPVL	13.33
C57BL/6 murine model of CCl_4 plus PPVL	12.50
TAA plus PPVL induced model in Piezo1$^{flox/flox}$ mice	14.29
CCl_4 plus PPVL induced model in Piezo1$^{flox/flox}$ mice	13.33
TAA plus PPVL induced model in Piezo1$^{\triangle EC}$ mice	8.33
CCl_4 plus PPVL induced model in Piezo1$^{\triangle EC}$ mice	14.29

Supplementary Fig. 1

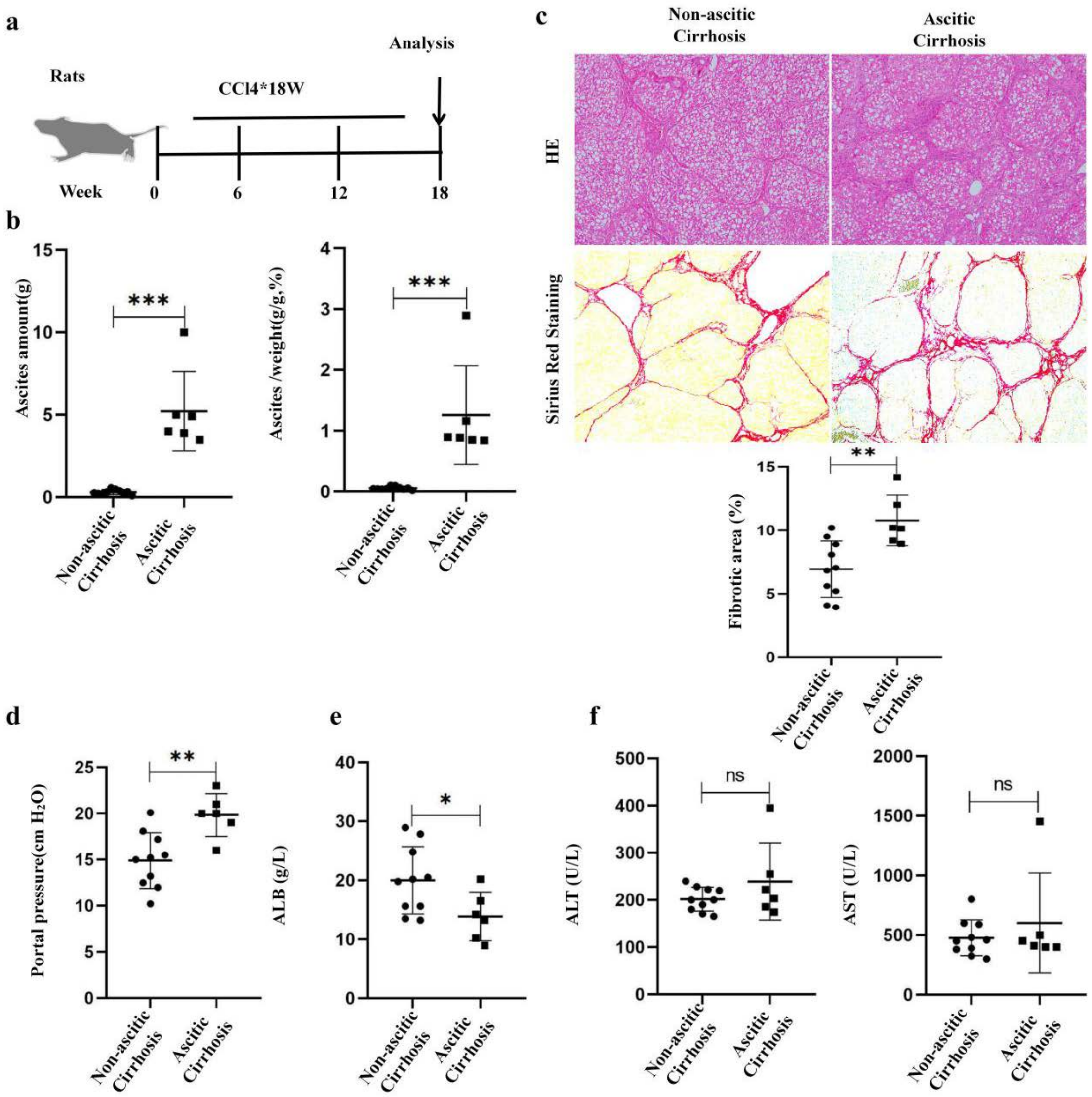

Supplementary Fig. 1 Rat model of liver cirrhosis induced by CCl_4 administration

a. Experimental protocol for the establishment of rat model of liver cirrhosis induced by CCl_4 administration; CCl_4, carbon tetrachloride;

b. ascites amount in cirrhotic rats;

c. representative images of hepatic H&E staining and collagen deposition; the percentage of fibrotic area was quantified by Sirius red staining (right panel)

d. portal pressure in cirrhotic mice;

e. serum albumin concentration in cirrhotic rats;

f. serum ALT, AST levels in cirrhotic rats; ALT, alanine aminotransferase; AST, aspartate aminotransferase; ** P<0.01, *** P<0.001, ns, no significant.

Supplementary Fig. 2

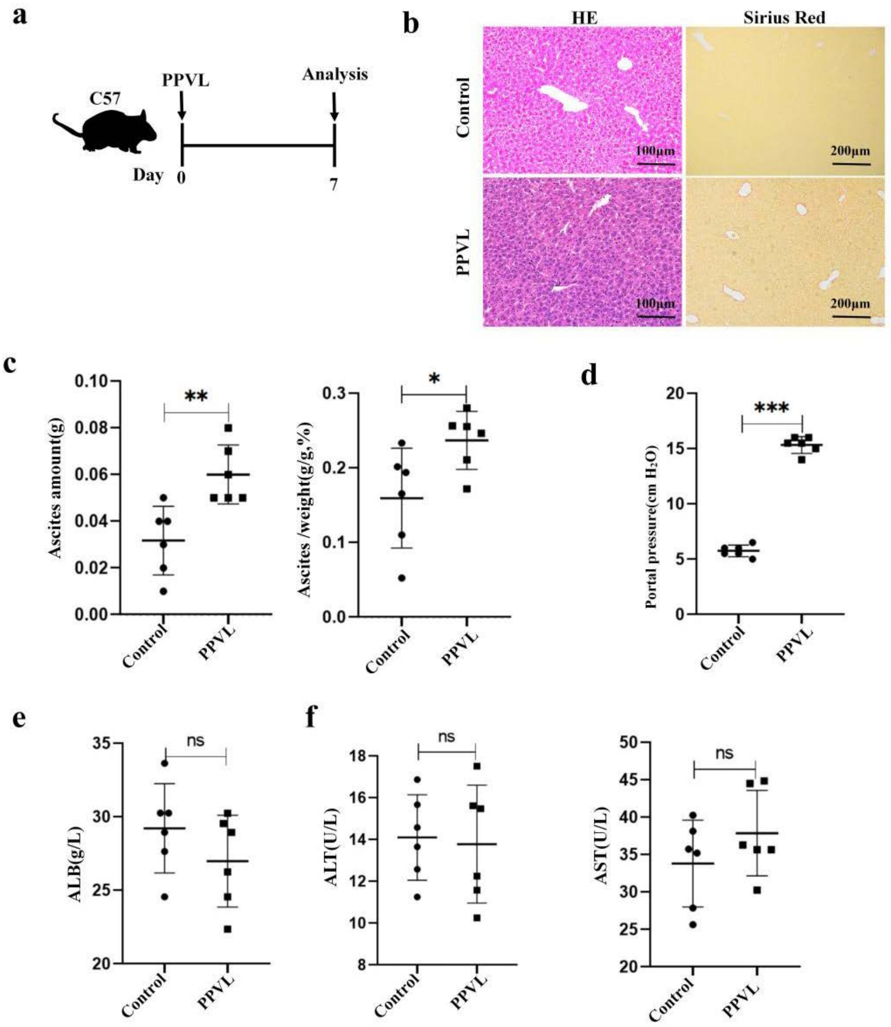

Supplementary Fig. 2 murine model of portal hypertension was established through partial portal vein ligation (PPVL).

a. Experimental protocol for the induction of portal hypertension without cirrhosis by PPVL;

b. representative images of hepatic H&E staining and Sirius red staining;

c. portal pressure in the mice which received PPVL.

d. ascites amount in the mice which received PPVL; left panel: ascites amount; right panel: ascites amount in body weight.

e. serum albumin concentration in the mice which received PPVL;

f. serum ALT, AST levels in the mice which received PPVL; ALT, alanine aminotransferase; AST, aspartate aminotransferase; * P<0.05, ** P<0.01, *** P<0.001, ns, no significant.

Supplementary Fig. 3

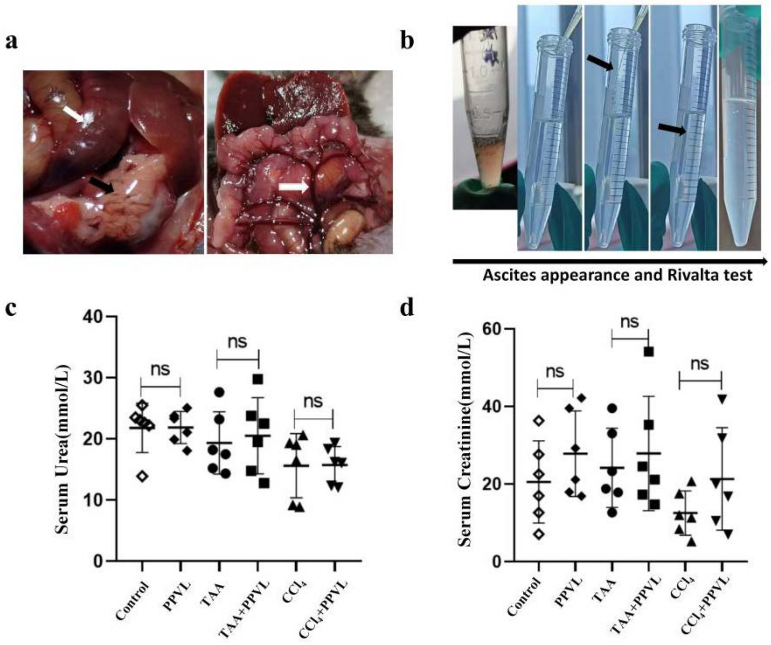

Supplementary Fig. 3 the pathological change of abdominal organ in murine model with liver cirrhosis and ascites, the result of Rivalta test and renal function.

a. Intestinal changes, the accumulation of peritoneal fluid and dilated portal vein in murine model with liver cirrhosis and ascites; left panel: intestinal congestion (white arrow), transparent peritoneal fluid on the omentum (black arrow); right panel: the dilation of portal vein (white arrow);

b. the appearance of ascites and Rivalta test for ascites collected from cirrhotic mice with ascites;

c. serum urea concentration in murine model with liver cirrhosis and ascites;

d. serum creatinine concentration in murine model with liver cirrhosis and ascites.

Supplementary Fig. 4

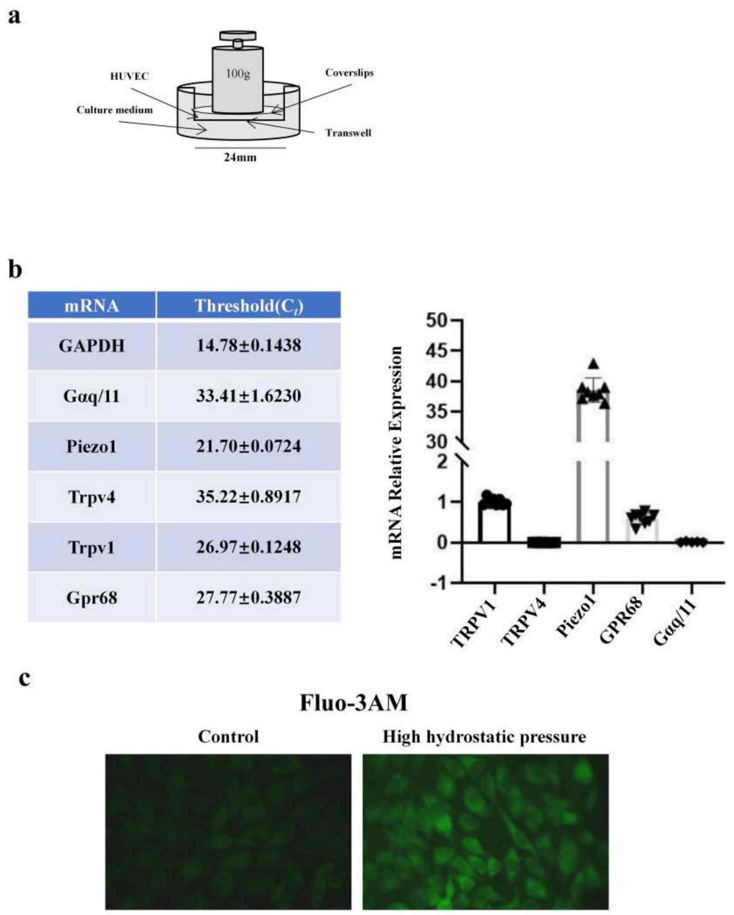

Supplementary Fig. 4 the activation of Piezo1 in HUVEC upon high hydrostatic pressure.

a. schematic diagram of the exposure of HUVECs to hydrostatic pressure *in vitro*;

b. quantitative RT-PCR determined the expression of several mechanosensitive ion

channels in HUVEC exposed to high hydrostatic pressure; Piezo1, piezo-type mechanosensitive ion channel component1; Gαq/11, G protein subunit alpha q11; Gpr68, G protein-coupled receptor68; TRPV1, transient receptor potential cation channel subfamily V member1; TRPV4, transient receptor potential cation channel subfamily V member 4;

c. calcium fluorescence of HUVECs revealing the activation of piezo1 in HUVEC upon high hydrostatic pressure.

Supplementary Fig. 5

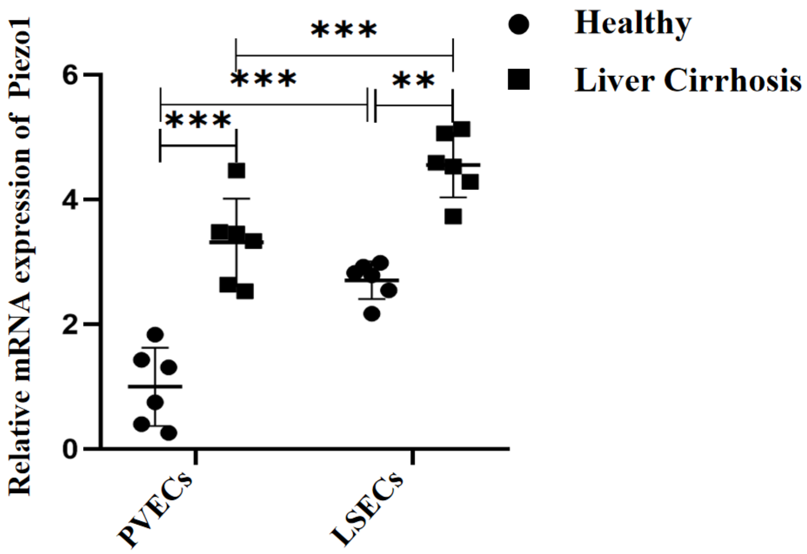

Supplementary Fig. 5. RT-qPCR showed that the expressions of piezo1 in peritoneal vascular endothelial and liver sinusoidal endothelial cells increased significantly in mice with liver cirrhosis than control mice.

Supplementary Fig. 6

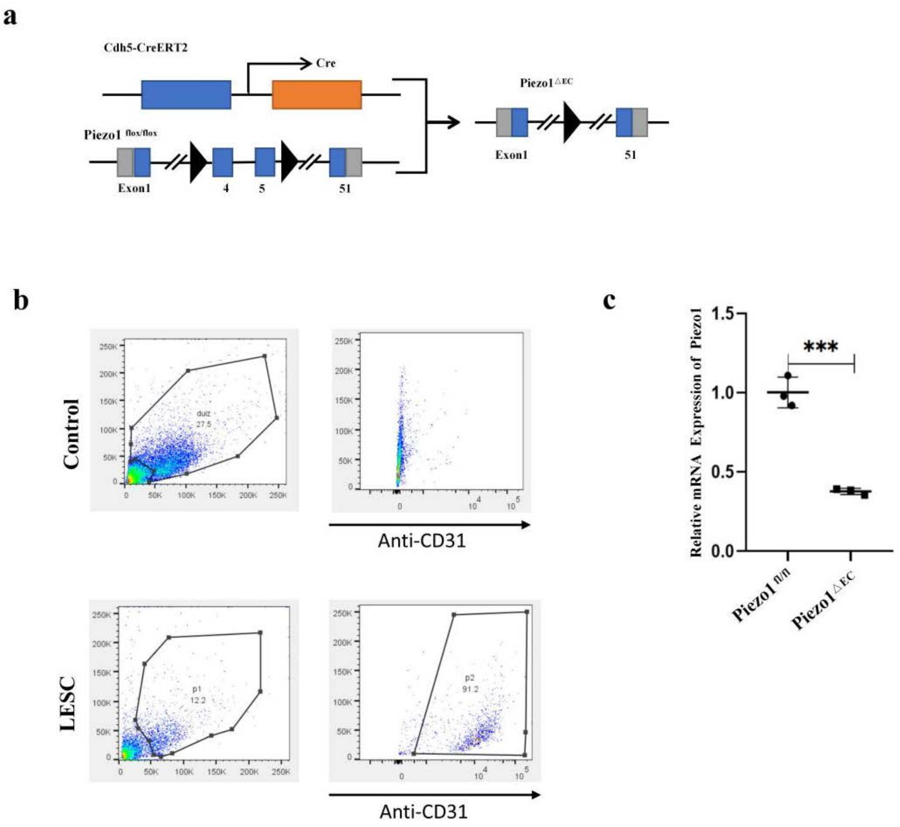

Supplementary Fig. 6 Generation of the mice with endothelial cell specific deletion of Piezo1(Piezo1$^{\triangle EC}$)

a**.** schematic diagram of the generation of the mice with targeted disruption of piezo1 in endothelial cells.； Piezo1$^{\triangle EC}$ mice were generated through breeding VEcadherin5-Cre mice with Pizol1floxed mice (Piezo1$^{fl/fl}$);

b. the purity of liver sinusoidal endothelial cells (LSECs) isolated through magnetic cell sorting;

c. piezo1 mRNA expression in endothelial cells obtained from Piezo1$^{\triangle EC}$ mice and Piezo1$^{fl/fl}$ mice.

Supplementary Fig. 7

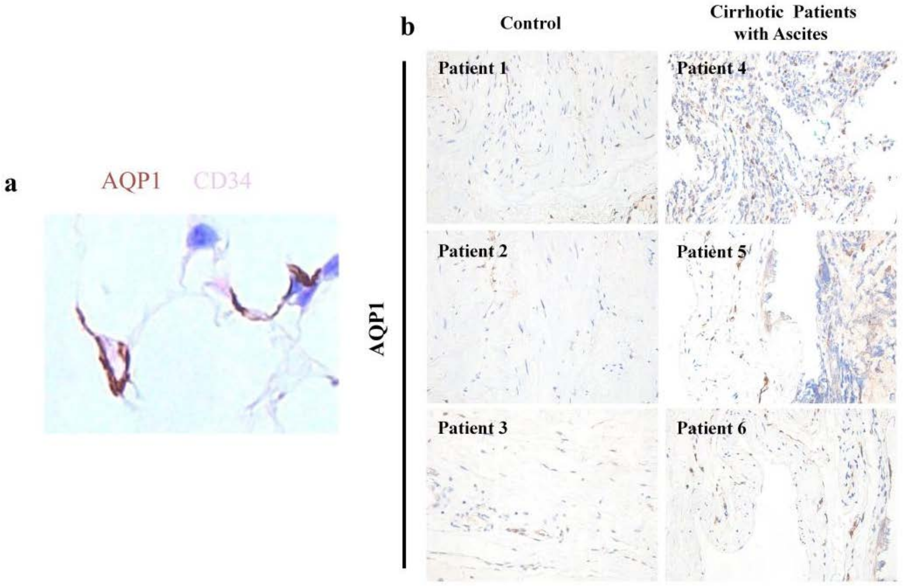

Supplementary Fig. 7 AQP1 expression in peritoneum of rat and cirrhotic patients with ascites

a. immunostaining of AQP1 and CD34 (vascular endothelial marker) showing AQP1 was predominantly expressed on microvascular endothelium in peritoneum of the rats.

b. increased expression of AQP1 in peritoneum was observed in cirrhotic patients with ascites.

Supplementary Fig. 8

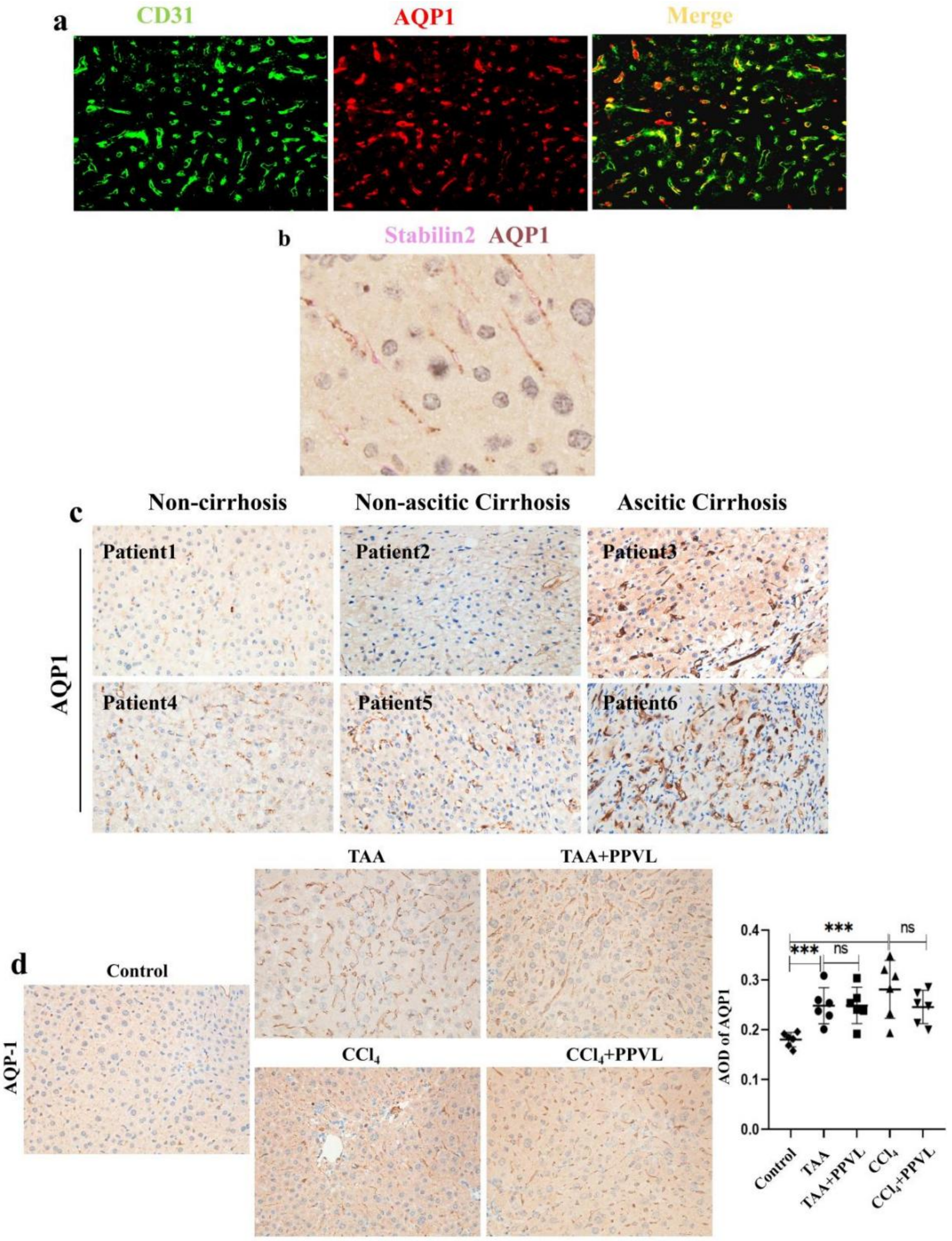

Supplementary Fig. 8 AQP1 expression in liver sections collected from cirrhotic mice and cirrhotic patients.

a. immunofluorescence of endothelial cell markers (CD31) and AQP1 demonstrated AQP1 was predominantly expressed in endothelial cells of murine liver tissues;

b. multiplex immunohistochemical (mIHC) results of Stabilin2 (LSEC-specific marker) and AQP1 revealed AQP1 in the murine liver is predominantly expressed in LSECs.

c. increased expression of AQP1 in liver tissues was observed in cirrhotic patients with ascites compared with control patients and cirrhotic patients without ascites.

d. AQP1 expression in liver tissues collected from cirrhotic mice with or without PPVL.

Supplementary Fig. 9

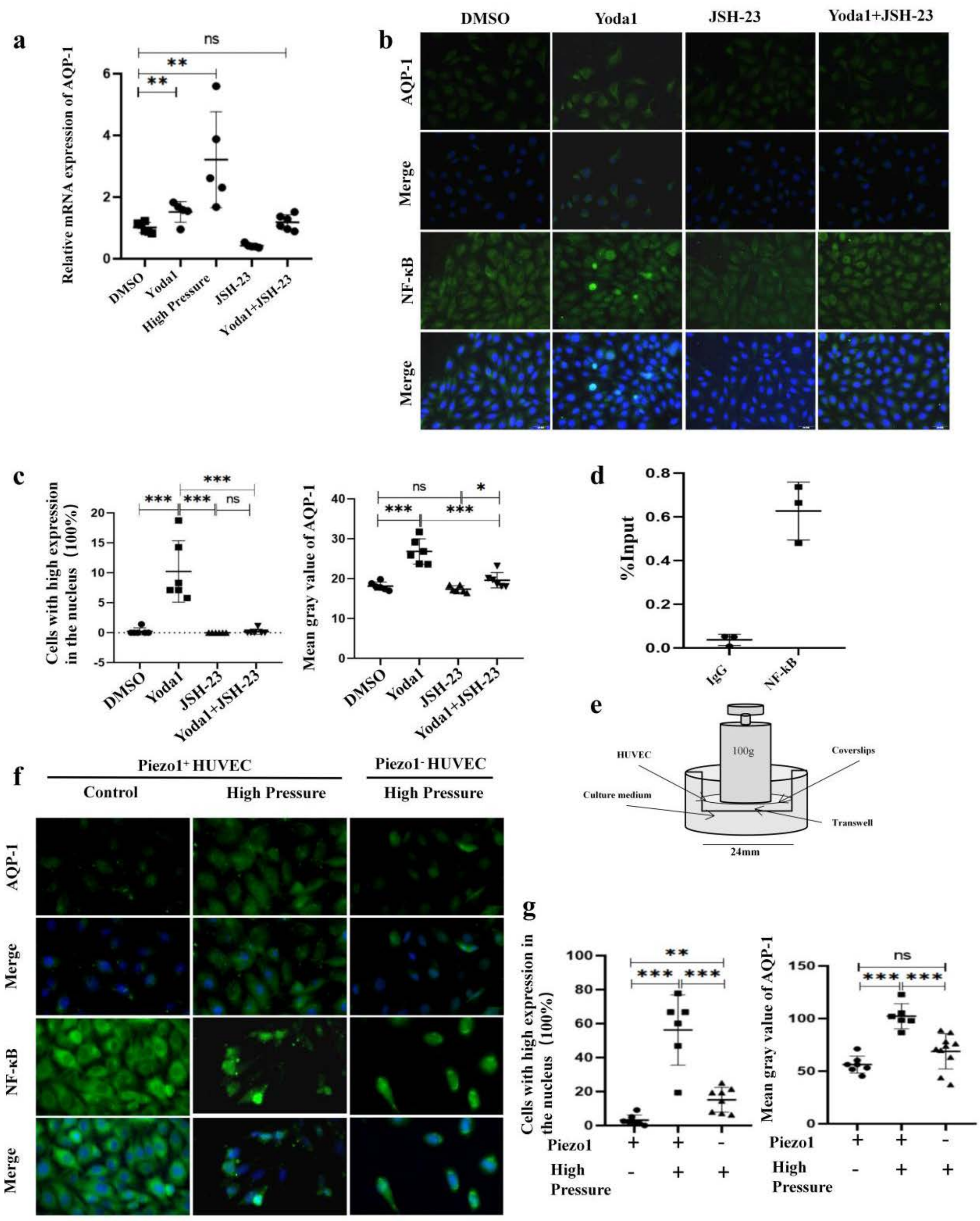

Supplementary Fig. 9 Piezo1 increased AQP1 expression in HUVECs through NF-κB.

a. the relative mRNA expressions of AQP1 in HUVECs treated with Yoda1(a Piezo1 agonist), high pressure, and JSH-23 (an inhibitor of NF-κB nuclear translocation);

b. the translocation of NF-κB and AQP-1 protein expression in HUVECs treated with Yoda1(a Piezo1 agonist), high pressure, and JSH-23 (an inhibitor of NF-κB nuclear translocation);

c. the statistical data of translocation of NF-κB (left panel) and protein expression of AQP-1 (right panel) in HUVECs exposed to Yoda1 and JSH23;

d. ChIP assay. HUVECs were treated with 1% formaldehyde to crosslink chromatin and subjected to immunoprecipitation using anti- NF-κB, with preimmune IgG as a control. The precipitated chromatin fragments were further analyzed by qRT-PCR using AQP1 primer;

e. schematic diagram of the exposure of HUVECs to pressure in vitro;

f. the translocation of NF-κB and AQP-1 expression in HUVECs exposed to high pressure;

g. the semi-quantitative analysis of translocation of NF-κB (left panel) and AQP-1 (right panel) in HUVECs exposed to high pressure.

* $P<0.05$, ** $P<0.01$, *** $P<0.001$. ns, no significant.

Supplementary Fig. 10

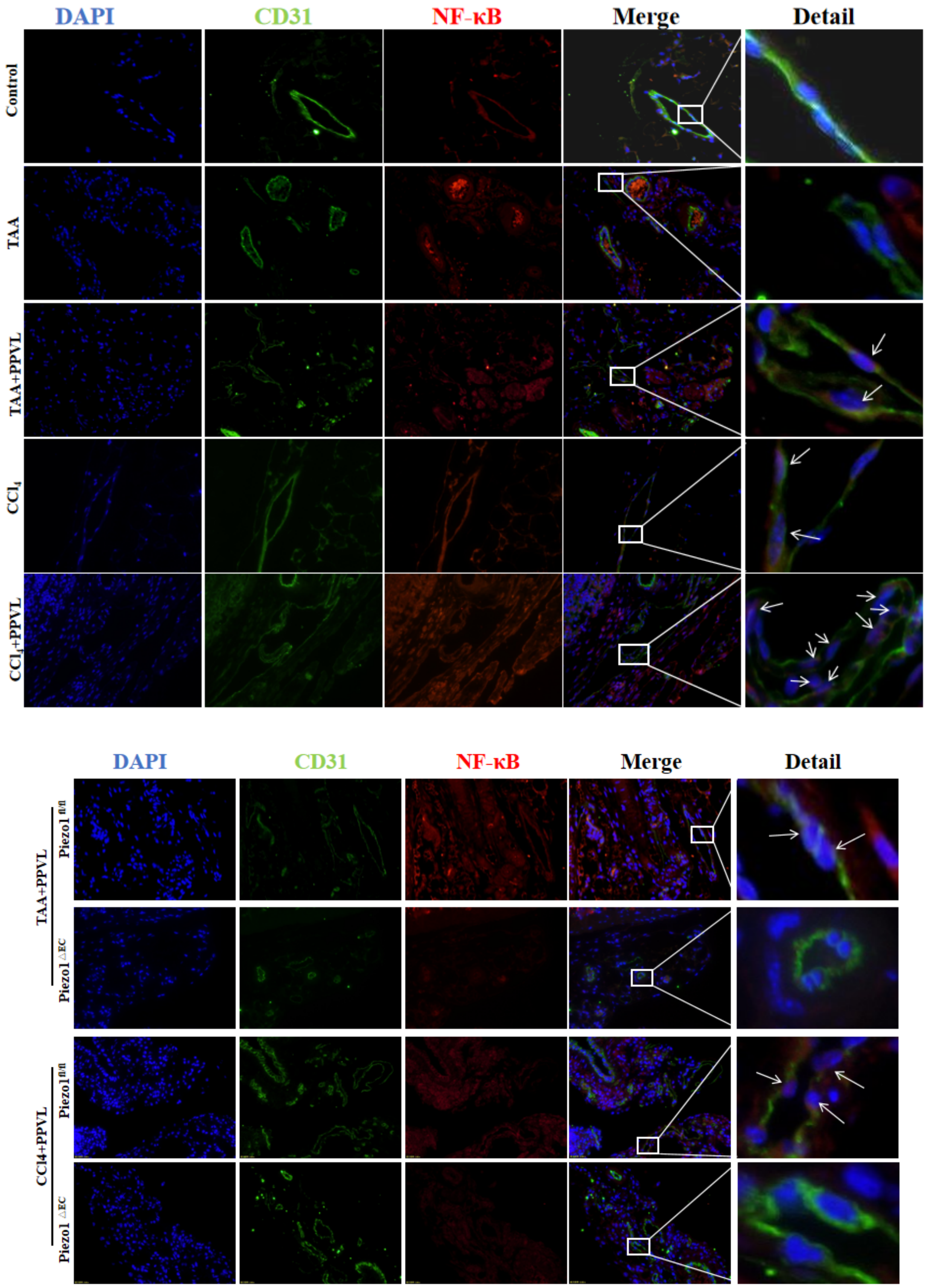

Supplementary Fig. 10 In vivo immunofluorescence of the intracellular distribution of NF-κB. (white arrow: NF-κB in the nucleus).

Supplementary Fig. 11

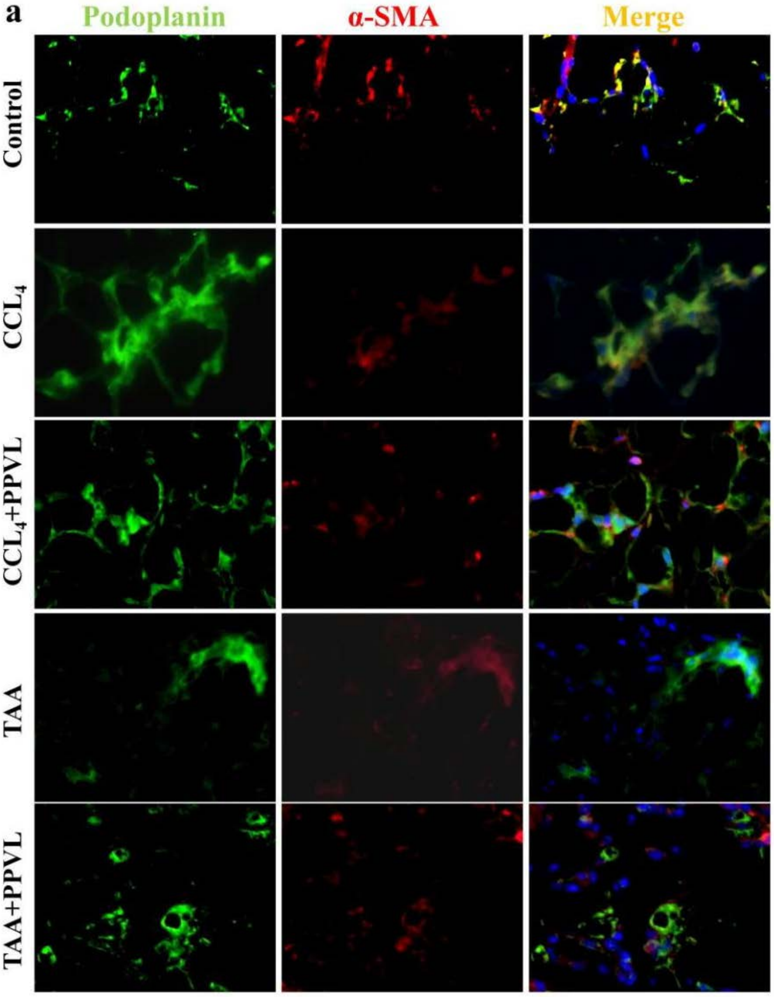
a
Podoplanin
α-SMA
Merge
Control
CCL4
CCL4+PPVL
TAA
TAA+PPVL

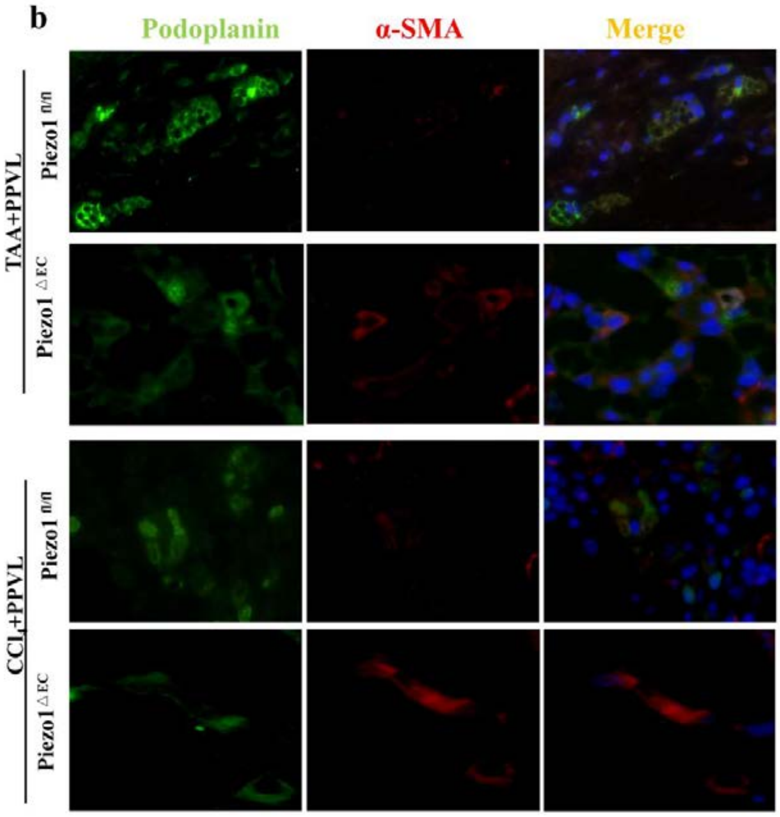

Supplementary Fig. 11 In vivo immunofluorescence of lymphatic vessels （Podoplanin） and smooth muscle cell (SMC， α-SMA) coverage.

a. decreased smooth muscle cells (SMCs) coverage surrounding lymphatic vessels were observed in cirrhotic mice with ascites compared cirrhotic mice without ascites.

b. the SMC lymphatic coverage increased in Piezo1△EC mice compared with the Piezo1flox/flox mice.

References:

1 Slim, K. *et al.* Methodological index for non-randomized studies (minors): development and validation of a new instrument. *ANZ J Surg* **73**, 712-716 (2003).

2 Wang, Y. *et al.* Ephrin-B2 controls VEGF-induced angiogenesis and lymphangiogenesis. *Nature* **465**, 483-486 (2010).

3 Xu, T. *et al.* Myofibroblast induces hepatocyte-to-ductal metaplasia via laminin-αvβ6 integrin in liver fibrosis. *Cell Death Dis* **11**, 199 (2020).

4 Domenicali, M. *et al.* A novel model of CCl4-induced cirrhosis with ascites in the mouse.

J Hepatol **51**, 991-999 (2009).

5 Getachew, Y., Cusimano, F. A., Gopal, P., Reisman, S. A. & Shay, J. W. The Synthetic Triterpenoid RTA 405 (CDDO-EA) Halts Progression of Liver Fibrosis and Reduces Hepatocellular Carcinoma Size Resulting in Increased Survival in an Experimental Model of Chronic Liver Injury. *Toxicol Sci* **149**, 111-120 (2016).

6 Zipprich, V. B., Nilius, R., Baust, G., Bunge, H. J. & Pauer, H. D. [Studies on the functional ability of swine liver perfused with human blood in machine recirculation attempt. Experiment design, elimination of indocyanine green, galactose, bilirubin and ammonia, synthesis of urea, bile secretion]. *Z Gesamte Inn Med* **31**, 364-368 (1976).
